# Response of Benthic Fauna to Habitat Heterogeneity in a Shallow Temperate Lake

**DOI:** 10.3390/ani11092488

**Published:** 2021-08-25

**Authors:** Krystian Obolewski, Katarzyna Glińska-Lewczuk, Marcin Sidoruk, Monika Magdalena Szymańska

**Affiliations:** 1 Department of Hydrobiology, University of Kazimierz Wielki in Bydgoszcz, 85-090 Bydgoszcz, Poland; szymanska.monika@ukw.edu.pl; 2 Department of Water Resources and Climatology, University of Warmia and Mazury in Olsztyn, 10-719 Olsztyn, Poland; kaga@uwm.edu.pl (K.G.-L.); marcin.sidoruk@uwm.edu.pl (M.S.)

**Keywords:** macroinvertebrates communities, freshwater fauna, aquatic morphometry, environmental parameters, bioassessment

## Abstract

**Simple Summary:**

Benthic fauna is an important element of the aquatic trophic chain, the basic element of biomonitoring. Our research was aimed at demonstrating the sensitivity of benthic fauna to the diversified morphometry of a single lake on the example of Lake Wicko (southern coast of the Baltic Sea). Our results show that lake morphometry plays a major role as a structuring factor for macroinvertebrates communities. Two segments of the lake, different in size and depth, show decreasing differences in the trophic state, abundance, diversity and number of indicator species of benthic fauna with the depth gradient. The greatest differences in abundance were observed in the shallowest zone of the bottom (eulittoral), where the chironomids differed the most. In the sublittoral of both lake segments, a simplified structure of benthic communities was found. Differences between the two segments within the intermediate zone (infralittoral zones) were recorded for mollusks and large crustaceans and the Oligochaeta/Chironomidae abundance ratio. The feeding groups were dominated by shredders in the eulittoral and infralittoral of the deeper lake segment. We recommend testing benthic macroinvertebrates in lakes with different morphometrics individually for each depth zone.

**Abstract:**

We investigated the response of benthic macroinvertebrates in the eulittoral, infralittoral, and sublittoral zones, in two segments of the freshwater Lake Wicko on the coast of the Baltic Sea. Our results showed that the morphometry of lakes plays a major role as a factor structuring the macroinvertebrates communities. Two parts of the lake, different in size and depth, show decreasing differences in the trophic state, abundance, diversity and number of indicator species of benthic fauna with the depth gradient. The most significant differences were observed between the littoral zones of both segments. Similar environmental conditions in the sublittoral zones corresponded to the simplified structure of the benthic macroinvertebrates communities. In the infralittoral zone, the most significant differences between the two segments, were recorded for mollusks and large crustaceans as well as the Oligochaeta/Chironomidae abundance ratio. In the sublittoral zone, the diversity of chironomids differed most strongly. Lower species diversity was found in the part of the lake with a slight depth decrease. Shredders reached significantly higher values in eulittoral and infralittoral of the deeper lake segment. Average Score Per Taxon increased with a depth gradient. We recommend testing benthic macroinvertebrates in lakes with different morphometrics individually for each depth zone.

## 1. Introduction

Natural changes of lakes and the associated gradual increase in eutrophication are major causes of ecological degradation in terms of aquatic biodiversity. Such changes are primarily due to the accumulation of auto- and allochthonous particulate substances, which affect all the processes of exchange of energy and matter between lake components (bottom, water, shoreline) and their surroundings [1].

Shallow polymictic lakes reach an advanced stage of development because almost their entire water volume takes part in most of the energy processes and phenomena, along with a relatively large surface area of active bottom sediments. Such processes are typical of coastal lakes connected with, or isolated from, the sea [2,3]. They are large and shallow, so changes in their morphometric parameters are some of the most important abiotic indicators of evolution of those ecosystems [3]. The lake basin morphometry is usually characterized by distinctly different habitat conditions in the littoral (coastal zone), open water (pelagic zone) and bottom (benthic zone) [4]. Among them benthic macroinvertebrates make up the dominant group of biomarkers (70%) [5]. These bottom-dwelling organisms are ceaselessly exposed to environmental changes and express their sensitivity towards varying degrees of stress over various time scales by their diversity, abundance, physiology, morphology, and behavior [6]. The benthic macroinvertebrates is suitable for assessment because they are mainly sessile organisms, thus meeting the assumptions of ideal indicator organisms. Furthermore, they promptly respond to any environmental changes and overall degradation of lakes [7,8]. Their location on the surface of bottom deposits, in spaces of interstitial sediments or dug-up burrows, constitutes an important factor for trophic relations [9]. Benthic macroinvertebrates mediate trophic transmission from the first level of consumers in the detritus food chain to the upper levels of consumers, and ultimately predators [10]. They produce a morphologically and behaviorally diversified range of mechanisms of exploitation of the available categories of trophic resources, in the form of coarse organic particles (CPOM, particles > 1.0 mm), fine organic matter (FPOM, particles > 0.45 μm and < 1.0 mm) and living organisms. In this way, benthic macroinvertebrates can be subdivided into functional feedings groups (FFGs) [11].

The availability of particular types of food in different zones of a lake is significantly diversified due to a depth gradient from the littoral to the deep profundal [11]. This feature enhances the opportunity for particular taxa to settle down. Considering both shallower (eulittoral) and deeper (infralittoral) subzones in a lake, the littoral is characterized by a high variability of light, oxygen, and temperature, but also a rich food base exploited by a diverse benthic fauna classified as distinct FFGs. In the sublittoral zone, the living conditions for benthic fauna deteriorate due to a decrease in temperature, oxygen saturation, low light exposure, and lower food variety [12]. This results in a strong selection favoring organisms with wide ecological tolerance and low food selectivity.

Based on of the above, depth is a key predictor that helps to explain the distribution of the entire benthic community or its selected groups, e.g., chironomidae larvae [13,14]. Simultaneously, diverse benthic fauna can develop in the littoral of a deep lake, for which the shallow depth is not a stressor [6,12,15].

Variability and availability of littoral habitats within the same lake, creating mosaics consisting of not overgrown soft muddy bottoms and dense rush vegetation cover, require an adaptation of sampling and proper measurement methodologies. The deviation from methodically unified sampling protocols, benthofauna studies require optimally adapted research techniques for individual zones within the same lake. This approach results from the site-specific structure of benthic organisms in the eulittoral, infralittoral, and sublittoral zones, in which the organisms may differently react to natural or anthropogenic stressors. As an alternative approach, a macroinvertebrate functional analysis based on FFG ratios (dimensionless ratios are relatively independent of sample size) may be used [16,17] While taxonomic identification has been the basis of macroinvertebrate analysis, functional analysis offers an additional tool that allows for a much faster analysis that can be performed for each morphological zone of a lake. This hybrid approach, supported by statistical techniques, allows for assessing both the current lake condition and the directions of future succession of the lake [8]. In this context, benthic fauna may be also applied to the assessment of hydro-morphological changes in any aquatic ecosystem [12,18]. A comprehensive approach is possible, indicating not only the current state of lakes, but also the direction of their future changes [8,15]. For example, such an approach is necessary in the case of shallow lakes with well-developed (i.e., complex) shorelines and varied depth structures. While shallow ecosystems lead to biological responses in the form of a drastic simplification of the food web in the pelagic and bottom zones, wide depth gradients in a single lake favor habitat heterogeneity supporting the development of diverse groups of macroinvertebrates with different environmental requirements [3,7,8,18,19].

In this study we compared the qualitative and quantitative structure of benthic invertebrate communities in the eulittoral, infralittoral, and sublittoral zones of the coastal Lake Wicko. Its basin consists of two clearly distinguished segments (eastern and western), which differ in size and depth gradients. We hypothesized that the abundance and diversity of macroinvertebrates are lower in the shallower segment of the lake due to its simplified morphological structure. The objectives of this study were: (i) to determine whether, on the basis of abiotic predictors, the two segments can be treated as distinct ecosystems; (ii) to assess the effects of the shallowing of individual segments of the lake of invertebrate communities in terms of taxonomic structure and its abundance; and (iii) to identify the metrics and indicator species that can be used to trace the results of morphometric changes in the lake.

## 2. Materials and Methods

### 2.1. Study Area

**Figure 1:**
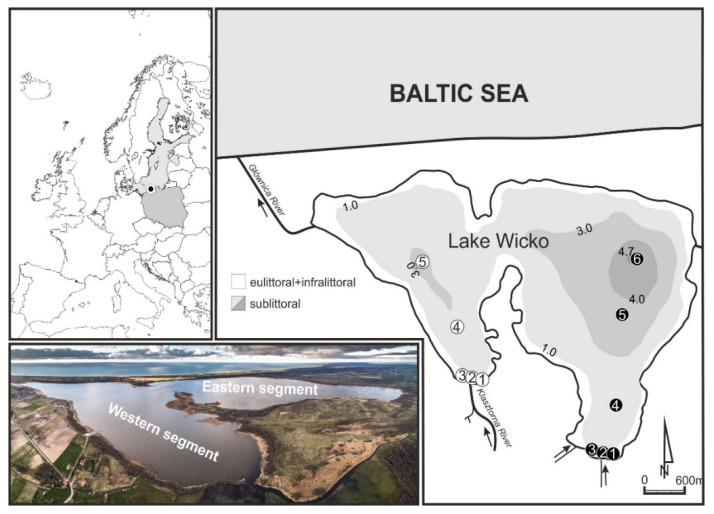
Location of sampling sites and morphometric cross-sections of Lake Wicko.

Lake Wicko, located in a crypto-depression on the southern coast of the Baltic Sea in north-western Poland (52°32′ N and 16°37′ E), is a relatively small water body (1031 ha). The freshwater coastal lake is separated from the sea by a wooded spit, crossed by the Głównica River, which links it with the sea in Figure 1.

The immediate neighborhood of the lake is dominated by arable lands (~60%), whereas the remaining catchment area is covered by forests, meadows, and pastures. The lake shoreline is morphologically well-developed, with much articulation. Two distinct segments of the lake (western and eastern) are separated by two peninsulas extending from the north and south. It is anticipated that because of progressive shallowing, two individual lakes will be formed in the near future. The lake is polymictic because of the shallow mean depth (h_mean_ = ~3 m), which enables mixing of the water from top to bottom, even during light winds. The shape of isobaths corresponds to the shape of the shoreline in both segments, but the eastern segment is deeper (h_max_ = 4.7 m). Between 1952 and 2014, the maximum depth of Lake Wicko decreased by 23% (from 6.1 to 4.7 m), the water table area shrank by 9% (from 10.6 to 9.8 km^2^) and lake water volume dropped by 17% (from 28,490 to 23,740 m^3^). This phenomenon was equally intensive in both segments of the lake: in the eastern segment, water volume declined from 22,684 to 18,928 m^3^, and in the western segment, from 5806 to 4813 m^3^. Simultaneously, lake shallowing was most rapid in the deepest zone (>3 m), as its area decreased by an average of 40% (35% in the eastern segment, and 44% in the western segment). Prevailing winds from the west contribute to greater lake depth in the eastern segment due to a more intensive horizontal eastward water movement as well as greater wave propagation and bottom sediment resuspension and erosion [20].

### 2.2. Environmental and Biological Samplings

Measurements of environmental parameters were made in parallel with biological sampling by using standard methods [21]. With the use of a multiparameter sonde (Aquaprobe^®^ AP-7000; Aquaread Ltd. Kent, UK) we measured in situ water temperature (Tw), electrical conductivity (EC), pH, oxygen saturation (DO%), salinity (Sal), and chlorophyll-*a* concentration (Chl-*a*). Water transparency was measured as Secchi depth (SD). The ionic composition of water samples was determined in the laboratory, where the cations (Na^+^, K^+^, Ca^2+^, Mg^2+^ and NH_4_^+^) and anions (NO_3_^−^, Cl^−^) were analyzed with the use of an ion chromatograph (881 Compact IC Pro: Metrohm, Herisau, Switzerland). Before analyses, samples were filtered with the use of 0.20-µm non-sterile filters (PTFT membrane, Merck Millipore Darmstadt, Germany), and then examined with the use of Metrosep C4 250/4.0 and Metrosep A Supp 5 250/4.0 columns with Metrosep C4 Guard/4.0 and Metrosep A Supp 4/5 Guard 4.0 pre-columns, respectively. Concentrations of total phosphorus and phosphates (TP and PO_4_^3−^-P) were determined in the laboratory with the use of spectrophotometry.

Based on Secchi depth data, chlorophyll *a*, and total phosphorus concentrations, we assessed trophic state using the Carlson trophic state index (CTSI), where TSI = Trophic State Index: CTSI = (TSI_SD_ + TSI_CHL_ + TSI_TP_)/3 after logarithmic transformation (Ln) [22]. Classification of trophic status of lakes was used after Alprol et al. [23].

Samples were collected three times, in both areas of the lake, during each year of the study (2014 and 2015). We sampled in spring (mid-May), summer (beginning of August), and autumn (mid-October), from the eulittoral, infralittoral, and sublittoral zones (Figure 1). In total, 66 samples were collected: 30 in the western segment (6 in eulittoral, 12 in infralittoral and 12 in sublittoral), and 36 in the eastern segment (6 in eulittoral, 12 in infralittoral, and 18 in sublittoral). The differences in a number of sampling sites resulted from different morphometries of segments of the lake. Results for samples collected at the same depth were averaged in each segment of the lake. The biological material was collected using the hybrid method by using two techniques dedicated to sampling three lake zones: manual tube sampler for sediment cores (area 21 cm^2^) in the vegetated eulittoral and infralittoral, and Ekman bottom grab sampler (area 225 cm^2^) in the soft bottom in sublittoral. For the analysis of biological material from such a comparable area, it was assumed that one sample collected with the Ekman bottom grab (3 subsamples) equals 30 subsamples collected with the core sampler. Bottom sediments with macroinvertebrates were sieved through a net (pore size 0.5 mm), and preserved in 4% buffered formaldehyde.

### 2.3. Bioassessment

Macroinvertebrates were identified in the laboratory usually to the species level. Some Oligochaeta and small individuals of the order Diptera were identified at higher taxonomic levels. Taxonomic data were recorded as presence/absence, relative abundance, and diversity indices (Shannon *α*-diversity index and Pielou evenness index). Relative abundance of functional feeding groups (FFG) to the total abundance of macroinvertebrates: ratio of grazers to scrapers (Gra/Scr), collector–filterers (FF), gatherer–collectors (GatCol), shredders (Shr), and predators (Pred). The classification of organisms into individual FFGs is based, among others, on the Cummins’ study [16] and Rawer-Jost et al. [17], without consideration of Chironomidae larvae (not detected), which can obtain food from different courses. Such a functional approach has been used for a long time in lotic ecosystems, but recently it has been adapted to lentic ecosystems [8,18]. Nevertheless, the response of FFGs to relative changes in the trophic state of lakes and their decreasing depths is still poorly studied.

To verify the response of the invertebrate community to environmental predictors (lake depth gradient and segment of the lake), we listed the potential metrics based on density, diversity, percentage contributions of some benthic groups or functional feeding groups, as well as the level of ecological tolerance [24]. In this way, according to the recommendations [11] we selected the following groups of metrics: (1) measure of species richness: number of all taxa (RICH_t), [25], the number of taxa of chironomid larvae (CHIR_t), [24,26], the number of EPT_t (Ephemeroptera + Plecoptera + Trichoptera larvae) [27]. (2) Percentage contributions of indicator groups: abundance of mollusks and large crustaceans in relation to the total macrofaunal abundance (%MolCru), which should decline with decreasing depth variation of the lake basin [25], and the abundance ratio of Oligochaeta to Oligochaeta + Chironomidae larvae (%OOC) [26]. (3) Biomonitoring: Biological Monitoring Working Party score (BMWP) and average score per taxon (ASPT). Although those measures are usually used to assess the ecological status of watercourses, they have also been adapted to lakes [5].

### 2.4. Statistical Analysis

To reduce the effect of absolute values, the abundances of the invertebrate densities were transformed (√(X + 1), rare species were downweighted and environmental data were log-transformed (log_10_ (X + l)) [28]. Multivariate statistical analyses were performed to examine spatial patterns in benthic community composition and environmental relationships. Additionally, correlation coefficients (r-value) between benthic macroinvertebrate abundance and depth in each basin were tested (with Bonferroni correction).

Using Bray–Curtis similarity [29], a matrix of associations between sample collection sites was generated on the basis of non-metric multidimensional scaling (NMDS) and similarity analysis (ANOSIM, 999 permutations). Based on these analyses, we assessed and tested differences in the Carlson trophic state index (CTSI) values, and diversity of macroinvertebrate communities in the two segments of Lake Wicko. Due to the low ‘noise’ of the dataset, we did not exclude from the analyses any taxa, even those rarely found in the samples. The NMDS was performed using Primer 7.0 software (PRIMER-e, Albany, Auckland, New Zealand).

To reduce co-linearity, we used a manual selection of parameters, choosing those that influenced the quality of the model at a level of at least 10%. We also applied the variance inflation index (VIF) to exclude co-linearity of the included variables [28]. The *p* values were determined using a Monte-Carlo permutation test [30].

Values of the metrics are shown in box plots, visualizing potential differences between the two segments of the lake, with a subdivision into three depth zones. Statistical significance of differences (*p* < 0.05) was verified using one-way ANOVA, whereas comparisons between the two segments and depth zones of the lake, employed the Mann–Whitney U test. At that stage, data were tested for normality (Shapiro–Wilk test) and homoscedasticity (Levene test), [28]. The response of the macroinvertebrate taxa and FFGs to environmental variables was analyzed using multivariate statistical analyses. To explain the density of taxa under study of macroinvertebrates and to associate them with environmental variables in segments (western and eastern) and zones (eulittoral, infralittoral, and sublittoral) we used a linear model of redundancy (RDA). Before RDA, we performed detrended correspondence analysis (DCA) to determine whether linear or unimodal ordination methods should be applied [28]. The scaling method was focused on the inter-species correlation. Both automatic and manual forward selection of environmental variables (Monte Carlo Permutation test, 999 unrestricted permutations) were used to test statistical significance (*p* < 0.05) of relationships between species indexes and environmental variables. Seasons were introduced as three dummy variables (spring = 1, summer = 2, and autumn = 3). Simultaneously, by eliminating factors that were not significant for the model, we selected a suitable subset of significant explanatory variables representing significant relationships between environmental variables and invertebrate taxa or biological indices. The specificity of habitats was shown on the basis of defining indicator organisms, and determination of indicator value (IndVal) based on the abundance of the *i*th group of macroinvertebrates in relation to *j*th depth zones [31]. The procedure was performed using CANOCO 5.10 (Microcomputer Power, Ithaca, US) and PC-ORD 6.0 (MjM Software, Gleneden Beach, Oregon, US) software.

## 3. Results

### 3.1. Environmental Conditions

The measured physicochemical parameters of water in Lake Wicko are shown in Table 1 and Table S1. Analysis of differences between the two segments of the lake indicated that they differ significantly in water quality (ANOSIM: *R* = 0.71, *p* = 0.009). Water in the western segment of Lake Wicko showed higher values of the physicochemical parameters than in its eastern segment, except for SD, *T_w_*, pH, DO%, and Ca^2+^. The analysis of differences in physicochemical properties of water between the depth zones in both segments of Lake Wicko showed that the largest contrasts were in concentrations of Chl-*a*, Na^+^, and Cl- (*p* < 0.0001), which were significantly higher in the western segment of the lake. Oxygen saturation differed significantly among measurement sites (*p* = 0.0001). The mean concentration of dissolved oxygen (DO%) in the western segment of the lake was 10% higher than in the eastern segment. Significant differences were also noted for visibility, pH, and K^+^ concentrations (*p* = 0.001).

Higher values of transparency and pH, were recorded in the eastern basin, while K+ concentration was higher in the western segment. The average values of lake waters differed significantly (*p* = 0.01) across sites, and were 15–20% higher in the western basin than in the eastern basin. The segments of the lake differed significantly (*p* = 0.04) in terms of TP concentrations: the mean concentrations of TP were higher in the western segment (0.24 mg L^−1^) than in the eastern segment (0.20 mg L^−1^). CTSI values indicated an advanced trophic state throughout the lake. The western segment of Lake Wicko was hypertrophic based on CTSI regardless of the lake zone, while the deeper eastern segment of the lake was characterized as eutrophic in the infralittoral and sublittoral depth zones (Table 1).

**Figure 2:**
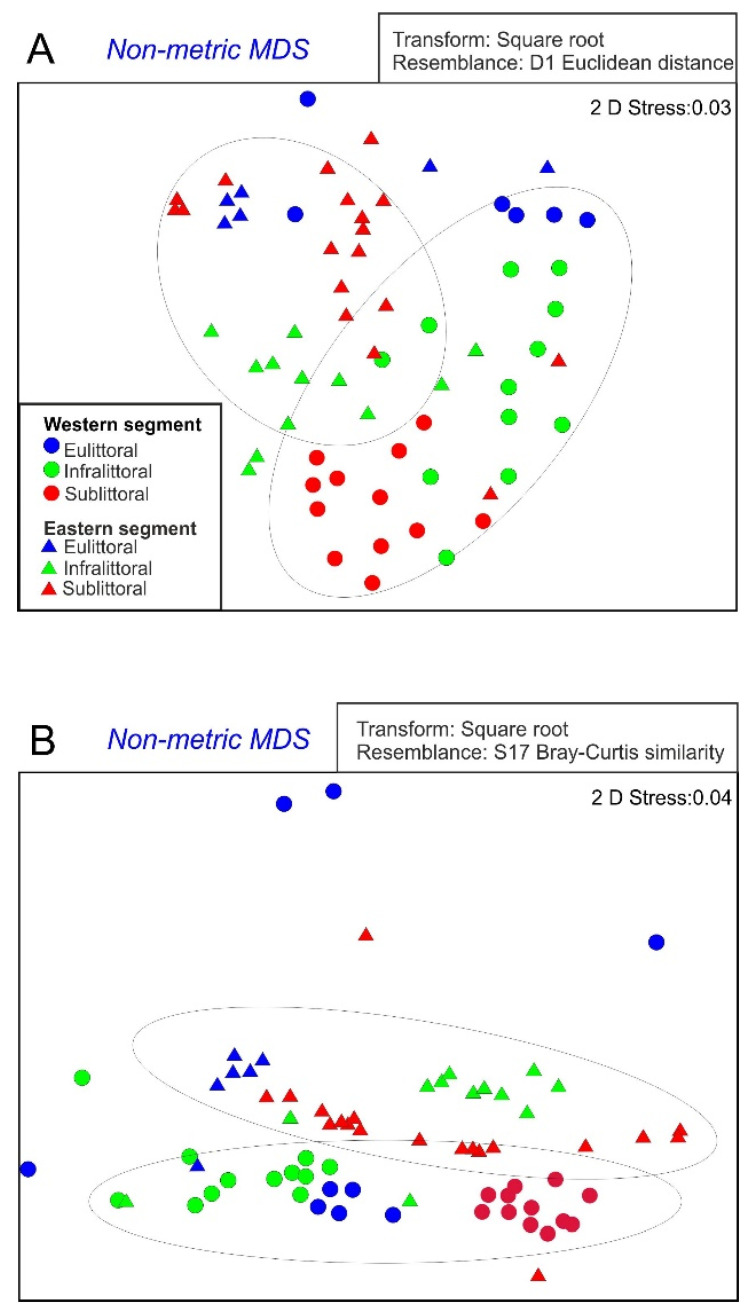
Results of non-metric multidimensional scaling (NMDS) ordinations showing the similarity of sampling sites in two segments of the study lake: (**A**) based on Carlson trophic state index (CTSI); and (**B**) based on the structure of benthic macroinvertebrate communities using invertebrate diversity.

The use of NMDS based on the trophic index CTSI Figure 2A, as well as the structure of benthic macroinvertebrate communities, Figure 2B clearly differentiated the two segments of the lake (ANOSIM: *R* = 0.77, *p* = 0.001 and *R* = 0.90, *p* = 0.001, respectively). The comparison of CTSI across individual depth zones in both segments of the lake revealed significant differences in the infralittoral (ANOSIM: *R* = 0.92, *p* = 0.001), and eulittoral (ANOSIM: *R* = 0.70, *p* = 0.002), whereas there were no differences in the sublittoral zone (ANOSIM: *R* = 0.07, *p* = 0.159).

The NMDS procedure, based on the abundance of benthic fauna community Figure 2B showed the greatest differences between both segments of Lake Wicko for communities (groups of species) determined on the basis of depth (ANOSIM: *R* = 0.98, *p* = 0.001). A clear difference between the two lake segments was also noticeable in the eulittoral (ANOSIM: *R* = 0.78, *p* = 0.02), while no significant difference was detected in the invertebrate community of the sublittoral zone (ANOSIM: *R* = 0.04, *p* = 0.12).

### 3.2. Macroinvertebrates Community Characteristics for Two Segments of the Lake

The measured abundance of benthic fauna in Lake Wicko is shown in Table 2 and Table S2. In the eastern segment, we identified 20 taxa in samples collected from the eulittoral, 25 from the infralittoral, and 13 from the sublittoral, while in the western segment we identified 15 taxa in the eulittoral, 22 in the infralittoral, and 9 in the sublittoral. Both the Shannon diversity index and the Pielou evenness index of macroinvertebrates were higher in the eastern segment, characterized by greater depth variation than in the western segment. Nevertheless, the highest values of both indices were recorded in the infralittoral of both segments of Lake Wicko (Table 2).

**Figure 3:**
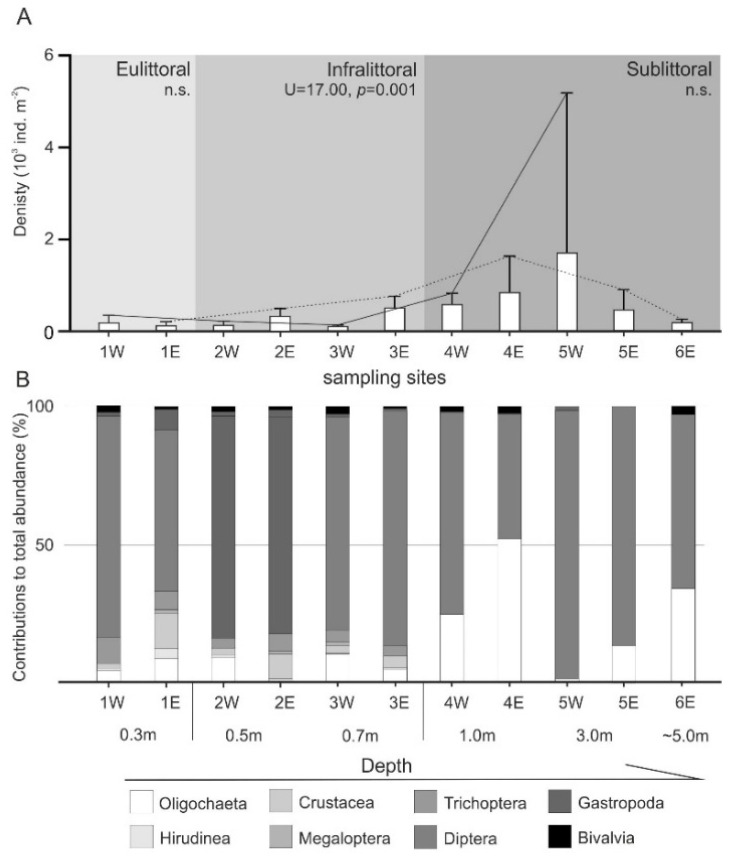
(**A**) Abundance of macroinvertebrates in different zones of two segments of Lake Wicko (W-western, E—eastern). The significance between the two lake segments (Mann–Whitney U test, n Bonferroni’s correction of *p*-values) is also reported, (n.s. = not significant). (**B**) Contribution of identified taxa in the total abundance of macroinvertebrates (%).

The abundance of macroinvertebrates in both segments of the Lake Wicko was similar only in its eulittoral zone. The infralittoral of the eastern segment was colonized by a 2 to 3-fold more abundant invertebrate community than the western segment. A similar pattern was observed in the sublittoral at a shallower depth (up to 1.0 m). At 3.0 m depth, invertebrate abundance was more than 3-fold higher in the western than in the eastern segment Figure 3A.

Diptera larvae contributed 44% to the total abundance of benthic infauna in Lake Wicko. Other groups were abundant only to the depth of 0.8 m, while the bottom was colonized nearly exclusively by Diptera and Oligochaeta larvae Figure 3B. Comparing individual depth zones of both segments of the lake, significant differences in the abundance of the same groups of macroinvertebrates were found in the infralittoral. Densities of Hirudinea and larvae of Trichoptera were higher (both at *p* = 0.03) in the infralittoral of western when compared to the eastern segment. An opposite relationship was observed for the abundance of Diptera larvae (*p* = 0.002) and Crustacea (*p* < 0.0001).

The distribution of FFG of macroinvertebrates showed significant differences in grazers/scrapers (Gra/Scr) between depth zones in Lake Wicko (*p* = 0.0001). Higher values of this group were observed in the western segment (Table 2). Among other values describing the trophic groups, there were significant differences in the presence of gatherer–collectors (GatCol), the share of which increased with depth in both segments. Collector-filterers (FF) were more numerous in the shallow water zone in the western segment, but the differences for the whole lake were not statistically significant. Similarly, insignificant differences in abundance were found for predators (Pred), which avoid the deepest zones (Table 2).

### 3.3. Environmental Conditions vs. Macroinvertebrates Structure

**Figure 4:**
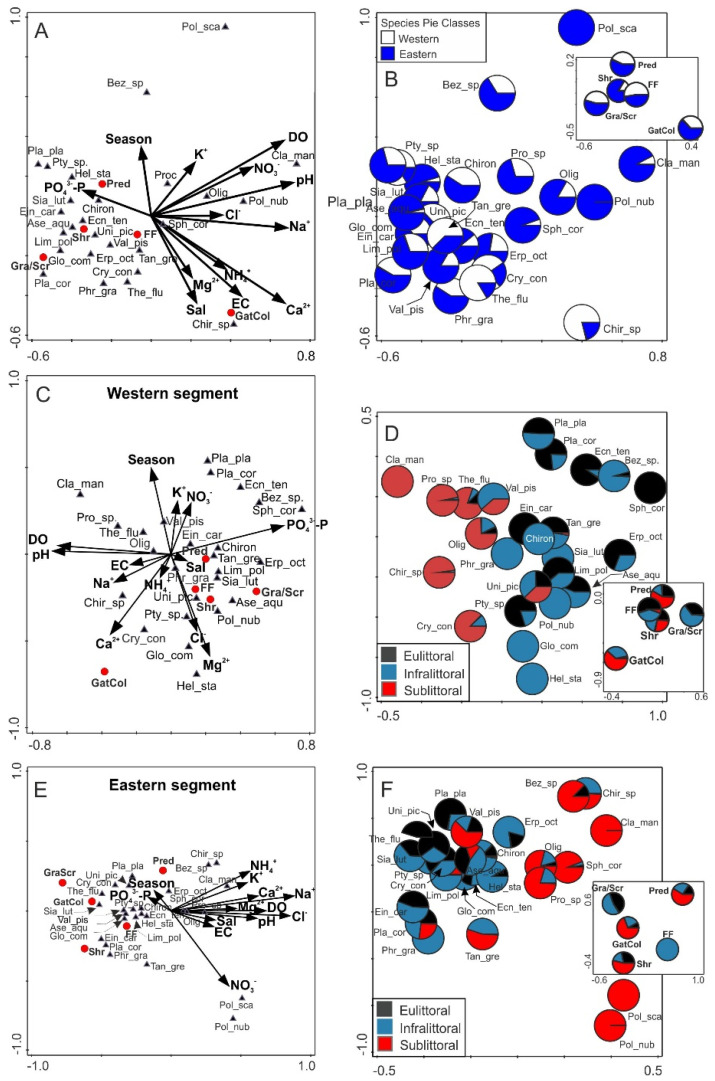
Results of triplot redundancy analysis (RDA) performed on percentage contributions of benthic macroinvertebrates taxa, functional feeding groups for environmental variables (**A**,**B**), in two segments (**C**,**E**) and their three depth zones (**D**,**F**) in the Lake Wicko (*p* < 0.05). B–Relative values of macroinvertebrates taxa and functional feeding groups in pie charts in relation to segments of lake, D and F–Relative values of macroinvertebrates communities and functional feeding groups in pies charts in relation to zones in each segments of lake. Code of taxa: Olig-Oligochaeta, Hirudinea: Erp_oct-*Erpobdella octoculata*, Glo_com-*Glossiphonia complanata*, Hel_sta-*Helobdella stagnalis*, Crustacea: Ase_aqu-*Asellus aquaticus*, Megaloptera: Sia_lut-*Sialis lutaria*, Trichoptera: Ecn_tel-*Ecnomus tenellus*, Phr_gra-*Phryganea grandis*, Lim_pol-*Limnephilus politus*, Diptera: Pty_sp.-*Ptychoptera* sp., Chiron-Chironomidae n. det., Ein car-*Einfeldia carbonaria*, Cla_man-*Cladotanytarsus mancus*, Chir_sp.-*Chironomus* sp., Cry_con-*Cryptochironomus conjugens*, Bez_sp.-*Bezzia* sp., Pro-*Procladius* spp., Pol_nub–*Polypedilum nubeculosus*, Pol_sca-*Polypedilum scalaeum*, Tan_gre-*Tanytarsus gregarius*, Gastropoda: Val_pis-*Valvata piscinalis*, Pla_cor-*Planorbarius corneus*, Pla_pla-*Planorbis planorbis*, The_flu-*Theodoxus fluviatilis*, Bivalvia: Sph_cor-*Sphaerium corneum*, Uni_pic-*Unio pictorum*; code of functional feeding groups: Gra/Scr-grazers/scrapers, FF-collector-filterers, GatCol-gatherer-collectors, Shr-shredders, Pred-predators.

In the model of RDA, component 1 (RDA1) explained 25%, while component 2 (RDA2) explained 16% of the total variance. All canonical axes were significant (pseudo-F = 1.5, *p* < 0.002). RDA1 was significantly related to nutrient concentrations, while RDA2 represented variation in water conductivity and salinity Figure 4A. Increased concentrations of PO_4_^3−^-P favored the increased density of Diptera larvae (Chironomidae, *Ptychoptera* sp.), Megaloptera (*Sialis lutaria*), Hirudinea (*Helobdella stagnalis*), and Gastropoda (*Planorbarius planorbarius*) and most of the FFGs. Higher salinity favored abundant *Chironomus* sp. larvae, while NO_3_^−^ favored *Tanytarsus gregarius* larvae Figure 4A. Benthic fauna were more abundant in the eastern segment of the lake. Only 15% of taxa (*Chironomus* sp., *T. gregarius Cryptochironomus conjugens* and *Teodoxus fluviatilis*) and collector-filterers were more abundant in the western segment of Lake Wicko Figure 4B.

The collinearity of abiotic predictors and benthic invertebrate taxa clearly differed between the two segments of the lake. In the RDA model computed for the western segment of the lake Figure 4C, RDA1 explained 33%, and RDA2 explained 21% of the total variance. All canonical axes were significant (pseudo-F = 2.6, *p* = 0.002). RDA1 was related to PO_4_^3−^-P, pH, and DO, while RDA2 to seasons, Mg^2+^ and Cl^−^. In the western segment, the increased concentrations of nutrients were accompanied by increased numbers of mollusks, predatory Trichoptera larvae (*Ecnomus tenellus*), and Diptera larvae (*Bezzia* sp.). Higher concentrations of Mg^2+^, Ca^2+^, and Cl^−^ favored gatherer-collectors Hirudinea (*H. stagnalis* and *Glossiphonia complanata*) as well as Diptera larvae (*Ptychoptera* sp. and *C. conjugens*) Figure 4C. The taxa of benthic macroinvertebrates inhabiting eulittoral and infralittoral zones were associated with RDA1, while macroinvertebrates (mainly predators and gatherer-collectors) preferring the sublittoral zone were associated with RDA2 Figure 4D. In the western segment of the lake, most taxa of benthic fauna reached the highest densities in the infralittoral, six of which appeared only within the depth range of 0.5–0.7 m. Two species appeared only in the eulittoral (*Sphaerium corneum, Einfeldia, e.g.*, *carbonaria*), and in the sublittoral (*Chironomus* sp. and *T. gregarius*). Only *Unio pictorium* Figure 4D had a comparable density distribution in individual zones of the western segment.

In the RDA model for the eastern segment of Lake Wicko, RDA1 explained 52% of variance, while RDA2 explained 18%. All canonical axes were significant (pseudo-F = 2.1, *p* = 0.002). RDA1 was significantly related to concentrations of Na^+^ and Cl^−^; RDA2 was associated with temperature and nutrient concentrations Figure 4E. Increased values of physicochemical predictors were associated with lower densities of benthic fauna, except for the abundance of Diptera larvae (*Polypedilum nubculosus* and *P*. *scalaeum*), which was associated with higher concentrations of NO_3_^−^ Figure 4E. The first component (RDA1) was associated with the abundance of benthic macroinvertebrates in the sublittoral zone, while RDA2 was associated with taxa inhabiting mostly eulittoral and infralittoral zones Figure 4F. In the case of *P. nubculosus*, a change in preferences for habitat depth was observed: from infralittoral in the western segment to sublittoral in the eastern segment. Correlations between individual taxa and depths for both segments have been shown in a Supplementary File (Table S3).

The taxa responsible for these differences were associated with the IndVal analysis (Table 3) and treated as good indicators of the status of the water body. Eleven taxa were identified as a characteristic of the ecological conditions in Lake Wicko. In the eastern segment, with greater depth variation, four characteristic taxa were distinguished in the eulittoral (*E. tenellus, L. politus, P. corneus* and *P. planorbius*), two in the infralittoral (*A. aquaticus* and *P. grandis*), and two in the sublittoral zone (Oligochaeta and *T. mancus).* The western segment, with smaller depth variation, was dominated by one order, namely, Diptera, characteristic of the eulittoral and infralittoral zones.

The distribution of biomarkers in the eastern segment of the lake was more homogeneous than in the western segment. Statistically significant differences between the two segments for the indicators in the infralittoral zones (U = 15.5, *p* < 0.001) were stated.

Among indices based on the diversity of macroinvertebrates, which we used for lake assessment, significant differences (*p* = 0.0001) were observed for total taxa (RICH_t) and number of taxa in the sensitive groups Ephemeroptera, Plecoptera, and Trichoptera (EPT_t), (Table 4). Both eulittoral and sublittoral zones in the eastern segment showed higher numbers of taxa (RICH_t), which directly influenced the values of biomonitoring indices BMWP and ASPT. Only the diversity of Chironomidae larvae (CHIR_t) was similar in the three zones of both segments of the lake.

The values of the %MolCru index were the highest in shallow waters of the eastern segment and decreased with increasing water depth. The share of Mollusca and Crustacea in benthic fauna density statistically differed across depth zones in both segments of the lakes (*p* = 0.0001). Differences in the distribution of the Oligochaeta and Chironomidae ratio (%OOC) were also statistically significant. Detailed information on the values of the bioassessment indicators is provided in the Supplementary Materials (Table S4).

## 4. Discussion

Benthic community in Lake Wicko is characterized by typical coastal shallow-water environments, namely low diversity and high dominance of a few opportunistic species [32]. The high variability of environmental conditions and the clear formation of two segments of the lake significantly influenced the spatial and seasonal distribution of benthic invertebrate species in different zones. This allowed establishing patterns of benthic fauna diversity forced by the depth gradients [8,19].

This study indicates that the composition of the invertebrate community in lakes is primarily determined by depth heterogeneity and degree of eutrophication. An example of Lake Wicko with clear division into two separate segments Figure 2, showed that biogeochemical processes within them are independent to a large extent. Environmental variables, although reflective of eutrophic conditions, predicted the invertebrate community structure in individual depth zones and the two segments of the lake. The greater depth heterogeneity of the eastern segment of the lake favored a higher abundance of macroinvertebrates (Table 2). Simultaneously, the depth gradient is an abiotic factor that determines habitat heterogeneity with respect to substrate type, thickness of sediments, access to light and nutrients, or water quality, as those factors directly affect the biotic components of the ecosystem [2,7]. This creates a situation of the structure of benthic fauna inhabiting one reservoir by two, hierarchical systems of environmental predictors. Greater heterogeneity of the depth of the eastern segment of the lake favored greater numbers of macroinvertebrates, but with the dominance of eurytopic taxa. In the case of Lake Wicko, such organisms are Chironomidae larvae, which dominate the shallow western segment and the shallow-water zone of the eastern basin Figure 3. According to many authors [33] these are typical phenomena, favoring the features of opportunistic species in nutrients in enriched or stressed habitats. Indeed, the levels of nutrients (especially phosphorus compounds) measured in the western basin exceeded those in the eastern basin (Table 1). Thus, the dominance of opportunistic traits observed in the western segment may be a response to natural phenomena (e.g., progressive succession) and human-induced stress (nutrient inflow). In our opinion, it is not easy to distinguish whether characteristics correspond to “natural” or “man-made” stress, or a combination of both [8].

### 4.1. Taxonomic Diversity and Functional Diversity

Many studies have shown the importance of macroinvertebrates inhabiting the littoral zone in the ecological assessment of lakes [6,18,34,35]. This zone creates a potentially better space for colonization than open water zones due to a more diverse mosaic of habitats. In Lake Wicko, the response of the invertebrate community to lake shallowing was less pronounced in the eastern segment (with the greater depth gradient), as evidenced by the higher species richness and values of diversity indices. In the deepest zone of the eastern segment of Lake Wicko, the major component of the fauna was highly eurytopic Oligochaeta [27], while in the sublittoral zone of the western segment, no characteristic species were distinguished.

The literature supports the idea that invertebrates inhabiting littoral zones, covered by macrophytes, are resilient to changes in environmental conditions of coastal lakes [2,8,36]. The plants supply the animals with food reserves, provide shelter, capture organic matter, and create interstitial space [27]. In Lake Wicko, the benthic macroinvertebrates of the infralittoral proved to be the most sensitive to lake shallowing, which is reflected in greater differences in taxonomic composition between the two segments of the lake. Notably, the eulittoral zone is generally characterized by lower nitrogen loads, but higher phosphorus loads than the sublittoral zone, which can be also linked to hydro-morphological changes in the water body [18,34,35]. For this reason, in the eulittoral zone, the response of invertebrates to the increase in trophic level in both segments of Lake Wicko is diminished. This dynamic complicates lake classification based primarily on littoral invertebrates, as the cited study attests to the complexity of relationships in the mosaic of habitats in the littoral zone of lakes. However, data on the structure of benthic fauna in the eulittoral and infralittoral are extremely important and confirm the significance of this zone for the assessment of ecological state [8,37].

Our results confirm the assumption that the correct use of taxa as indicators of ecological status should cover the whole depth gradient. Donohue et al. [37] indicated that the same taxa can be sensitive or tolerant to eutrophication depending on where they live. This suggests a need for complex assessment of the ongoing hydro-morphological processes, based on an analysis of benthic macroinvertebrates over the whole lake depth gradient. Additionally, in lakes with well-developed shorelines, their clearly distinguished segments should be analyzed separately. Environmental disturbances occur to some extent independently in individual segments of lakes, at different rates related to depth gradient (e.g., acidification or hydrostatic pressure on the littoral and deoxygenation in the sublittoral). In this context, detailed knowledge about communities living along the depth profile can make it possible to identify early signs of deterioration of the ecological status of lakes [8,38] and support early undertaking of remedial actions.

Our results also indicate a methodological recommendation that assessing the condition of lakes with well-developed coastlines and complex morphology divided into clearly distinguished segments should include a quantitative evaluation of benthic fauna based on the biological material collected from each depth zone.

### 4.2. Selected Metrics Based on Macroinvertebrates

Significantly higher values of water parameters Figure 2 in the western segment of the lake were linked to lower values of most biological metrics there (Table 4). Lang and Lods-Crozet [38], and Bazzanti et al. [8] indicate that chironomids are better indicators of changes in deeper lake zones than oligochaetes, because the latter are less mobile and more sensitive to deoxygenation. Simultaneously, the increase in the diversity of chironomids in littoral zones can be attributed to less environmental pressure in the shallow water zone, e.g., the relatively minor pressure of predation [13,18]. Results of this study show that the metrics based on the Chironomidae, dominating in coastal lakes, can be good tools for assessing the ecological status of deeper zones of lakes [26,37]. In this context, the Oligochaeta/Chironomidae abundance ratio, based on the two groups of benthic animals that are usually most abundant, has been used for assessing the trophic state in the deepest zones of water bodies, e.g., [26,39]. In case of Lake Wicko this indicator was significantly lower in the sublittoral of the eastern segment with the greater depth gradient. Surprisingly, in the infralittoral, it was significantly higher in the western segment, with a small depth gradient. These results could be caused by changes in the hydrological regime or advanced trophy in the shallow segment of the lake, forcing chironomid larvae to migrate [13,26,39].

The percentage contribution of mollusks and large crustaceans to the total abundance of benthic macroinvertebrates was significantly higher in the infralittoral in the lake segment with greater depth variation. In fact, this indicator increased also in the eulittoral and sublittoral zones, but not significantly. These patterns can correspond to other disturbances affecting the eulittoral (e.g., changes in water level) and deeper zones of the lake (e.g., deoxygenation in summer). This indicator seems sensitive to trophic level [8], and our study shows that it is also sensitive to differential trophic levels in two segments of the lake.

We stated that the spatial variation in CTSI of both segments of the lake clearly differentiated the functional structure of macroinvertebrates in the zones, which undoubtedly made the interpretation of the results more difficult. Among several metrics based on functional groups, the dominance of shredders in the lake zones is explained by uneven shallowing of the lake [20] and eutrophication, which is associated with it [40]. This effect can be caused by continuous expansion of macrophytes in the littoral zone, as plant remains are the main sources of food for this invertebrate group (e.g., for *A. aquaticus*). In the deepest zone, the increase in the abundance of chironomid shredders could be due to food quality, i.e., diversity of algal cells (from filamentous to unicellular) or taxonomic composition.

Finally, ASPT scores indicate significant differences between the depth zones in two segments of the studied lake. This result implies that most of the insect families sensitive to pollution prefer the littoral zone of the eastern segment, with a greater depth gradient. It could be expected that in the shallower and more eutrophic western segment, stenotropic invertebrates will concentrate in the shallow water zone. It must be remembered that the ASPT method was originally developed for lotic ecosystems, and its application to lakes is problematic [8,35], so further research is needed to verify its usefulness for assessment of littoral zones in lakes. Undoubtedly, conditions in lakes are complicated and specific to individual depth zones. The use of one metric, or focusing on one zone only, does not guarantee reliable results. It has been proved that among the investigated indicators, ASPT is a promising measure of eutrophication of individual lake zones and the degree of shallowing, whereas other biological diagnoses usually require general or specific identification of dominant groups, mostly chironomids and oligochaetes [26], which requires expert knowledge.

## 5. Conclusions

This study shows the possibility of using various metrics of benthic fauna for comprehensive assessment of trophic states of water bodies, with complex internal morphometrical structure, under the influence of eutrophication. Our study covered all zones of the lake, in which we used a hybrid methodology originally assigned to littoral and sublittoral zones. Our results show that differences in morphometry specifically affect the biota of each lake zone. In the littoral (especially the infralittoral), benthic macroinvertebrates react more strongly to an increase in nutrient concentrations in water as well as site-specific morphometry physical parameters of the lake bottom (uneven shallowing of individual segments of the water body). The metrics used here also indicate the importance of shoreline development, which can determine the degree of isolation of individual segments of the lake. Note that averaged results of an assessment of trophic state of a lake or its assessment based on a single measurement do not reflect the actual state of individual lake segments. Considering the above, it seems justified to quantify biological results using a zone-specific approach to give a complete picture of ecological status of a whole lake.

Considering that eutrophication and shallowing are currently the greatest threats to lakes, biological diagnoses of the intensity of those processes will soon become more important in limno-ecological analyses. Results of this study confirm that the process of successive deterioration of the ecological status of water bodies should be monitored in all zones on the basis of representative taxa and indices to reflect complex patterns. The selection of those biological tools should account for the morphometric specificity of individual water bodies, e.g., their depth gradient and shoreline development.

## Figures and Tables

**Table 1:** Annual mean or median* (±Standard Deviation) of physicochemical parameters of water at different depths in two segments of Lake Wicko (*n* = 66). Denotations: SD = Secchi depth; Tw = water temperature; EC = conductivity; DO = dissolved oxygen; TP = total phosphorus; CTSI = Carlson trophic state index; *p* = significance of differences between zones (one-way ANOVA, *p* < 0.05). ^E^ = eutrophic (54 < CTSI ≤ 74); ^H^ = hypereutrophic (CTSI > 74); according to Alprol et al. [23].

	Western Segment *n* = 30					Eastern Segment *n* = 36						*p*
Depth (m)	Eulittoral	Infralittoral		Sublittoral		Eulittoral	Infralittoral		Sublittoral			
	0.3	0.5	0.7	1.0	3.0	0.3	0.5	0.7	1.0	3.0	~5.0	
SD (m)	0.20(0.00)	0.39(0.04)	0.27(0.03)	0.31(0.06)	0.30(0.07)	0.20(0.00)	0.38(0.05)	0.32(0.10)	0.32(0.07)	0.34(0.09)	0.35(0.08)	<0.0001
*T_w_* (^o^C)	15.8(3.4)	14.7(4.5)	17.0(5.3)	16.5(4.9)	16.4(5.0)	18.5(5.4)	18.6(5.7)	16.0(4.8)	17.4(5.4)	16.4(4.4)	16.1(4.5)	0.75
pH *	8.09	8.13	8.56	8.70	8.76	8.18	8.15	8.68	8.71	8.63	8.56	0.0001
DO (%)	50.9(9.7)	57.6(17.2)	99.7(24.1)	106.3(20.6)	107.6(19.1)	61.7(10.0)	75.6(10.9)	103.4(18.4)	109.4(24.8)	100.3(16.5)	100.6(25.1)	<0.0001
EC (μS cm^−1^)	269(60)	277(54)	345(117)	299(138)	298(137)	185(24)	217(50)	289(135)	286(135)	289(136)	302(130)	<0.008
Salinity (PSU)	0.14(0.03)	0.10(0.01)	0.14(0.08)	0.12(0.09)	0.12(0.09)	0.08(0.03)	0.11(0.03)	0.12(0.08)	0.12(0.08)	0.12(0.08)	0.12(0.08)	0.28
NO_3_^−^ (mg L^−1^)	0.67(0.29)	0.60(0.30)	0.86(0.38)	0.65(0.24)	1.85(2.22)	0.23(0.08)	0.33(0.09)	0.79(0.43)	0.77(0.49)	0.89(0.48)	1.26(0.85)	0.18
NH_4_^+^ (mg L^−1^)	0.31(0.12)	0.47(0.28)	0.36(0.33)	0.28(0.13)	0.29(0.16)	0.16(0.08)	0.15(0.05)	0.37(0.35)	0.25(0.19)	0.32(0.22)	0.33(0.35)	0.15
PO_4_^3−^-P (mg L^−1^)	0.03(0.01)	0.02(0.01)	0.02(0.01)	0.02(0.00)	0.02(0.01)	0.02(0.01)	0.02(0.01)	0.01(0.01)	0.02(0.01)	0.02(0.01)	0.02(0.01)	0.04
TP (mg L^−1^)	0.67(0.13)	0.54(0.11)	0.64(0.35)	0.34(0.38)	0.31(0.39)	0.50(0.21)	0.46(0.18)	0.42(0.42)	0.56(0.30)	0.48(0.30)	0.47(0.31)	0.16
Chl-a (μg L^−1^)	151.7(59.4)	35.0(3.8)	6.5(1.0)	174.5(77.8)	36.2(30.2)	27.2(6.6)	38.3(9.4)	5.6(0.5)	43.1(19.4)	6.01(0.21)	6.07(0.26)	<0.0001
Cl^−^ (mg L^−1^)	52.4(6.5)	70.1(8.2)	70.3(25.6)	54.4(3.6)	55.4(3.4)	28.9(8.3)	36.9(4.8)	50.3(4.0)	51.1(2.4)	52.0(2.7)	51.7(2.0)	<0.0001
Na^+^ (mg L^−1^)	22.7(8.1)	24.1(8.1)	45.4(18.9)	33.6(3.7)	34.4(3.5)	16.7(3.2)	15.5(3.2)	31.3(2.9)	31.7(2.3)	32.0(2.2)	32.5(2.1)	<0.0001
K^+^ (mg L^−1^)	2.4(1.1)	2.1(0.6)	5.5(2.0)	3.8(0.7)	4.4(1.1)	1.9(0.3)	2.0(0.7)	4.1(1.2)	4.1(1.2)	4.0(1.2)	4.1(1.1)	0.001
Ca^2+^ (mg L^−1^)	18.0(2.9)	17.8(5.1)	22.5(6.5)	24.3(6.6)	19.4(5.5)	15.8(2.7)	16.0(3.2)	23.7(10.6)	25.6(15.8)	27.5(13.5)	26.2(12.3)	0.16
Mg^2+^ (mg L^−1^)	2.9(0.4)	3.1(1.2)	4.5(2.8)	3.0(2.2)	2.6(2.4)	2.2(0.6)	1.6(0.6)	3.1(1.9)	3.1(2.3)	3.1(2.2)	3.5(2.0)	0.35
CTSI	87(2) ^H^	78(2) ^H^	74(5) ^E^	79(8) ^H^	72(10) ^E^	79(4) ^H^	77(4) ^H^	69(8) ^E^	79(6) ^H^	71(5) ^E^	71(4) ^E^	

**Table 2:** Annual mean values of diversity indices and abundance (ind. m^−2^ ± Standard Deviation) and FFG (%) of the invertebrate community in different depth zones of two segments of Lake Wicko in 2014–2015; *p* = significance of differences between zones (one-way ANOVA, *p* < 0.05).

	Western Segment *n* = 30					Eastern Segment *n* = 36						*p*
Depth (m)	Eulittoral	Infralittoral		Sublittoral		Eulittoral	Infralittoral		Sublittoral			
	0.3	0.5	0.7	1.0	3.0	0.3	0.5	0.7	1.0	3.0	~5.0	
Richness of taxa	15	18	15	7	7	20	15	15	8	8	8	<0.0001
α-diversity	1.06(0.36)	1.09(0.39)	0.93(0.28)	0.81(0.35)	0.66(0.33)	1.75(0.39)	1.06(0.48)	1.12(0.43)	0.59(0.36)	0.95(0.26)	0.87(0.33)	<0.004
Evenness	0.56(0.20)	0.56(0.12)	0.53(0.11)	0.64(0.10)	0.75(0.22)	0.60(0.20)	0.36(0.15)	0.40(0.09)	0.56(0.30)	0.78(0.13)	0.80(0.10)	0.0001
Oligochaeta	8.6(9.6)	13.5(16.6)	8.3(5.4)	69.1(73.7)	19.8(26.6)	13.3(6.7)	1.2(1.7)	20.2(31.1)	437.0 (590.6)	61.7(47.2)	54.3(52.5)	<0.0001
Hirudinea	0.9(2.1)	2.1(4.1)	0.4(0.9)	0.0	0.0	5.8(5.8)	1.7(2.1)	4.4(3.9)	0.0	0.0	0.0	<0.0001
Crustacea	4.7(6.7)	3.9(8.8)	2.1(4.6)	0.0	0.0	19.2(13.6)	29.0(15.6)	20.8(15.1)	0.0	0.0	0.0	0.04
Megaloptera	0.0	0.2(0.5)	0.9(2.1)	0.0	0.0	2.0(4.0)	3.1(4.6)	0.4(0.9)	0.0	0.0	0.0	<0.0001
Trichoptera	19.5(19.0)	5.7(4.9)	3.7(6.2)	0.0	0.0	10.0(4.7)	20.1(21.0)	18.3(22.5)	0.0	0.0	0.0	0.02
Ptychoptera	26.8(33.0)	11.9(16.9)	1.6(2.1)	74.1(45.5)	66.7(55.9)	19.7(22.4)	16.6(37.0)	52.9(56.0)	254.3 (288.3)	24.7(22.1)	54.3(55.2)	0.07
Chironomidae	140.0(95.9)	105.5(40.9)	62.1(28.9)	130.9(130.7)	1575.3(3214.6)	65.7(21.9)	228.6(155.2)	364.4(208.0)	106.2(131.5)	363.0(384.3)	44.4(40.1)	0.08
Ceratopogonidae	0.6(0.9)	12.4(19.5)	0.0	0.0	0.0	3.2(7.2)	0.0	0.0	19.8(26.6)	0.0	2.5(5.5)	<0.02
Gastropoda	2.3(3.8)	2.5(2.7)	1.0(1.4)	1.2(2.8)	24.7(55.2)	11.3(12.2)	8.0(4.6)	4.3(9.5)	2.5(5.5)	0.0	0.0	0.34
Bivalvia	4.9(6.4)	2.7(2.4)	2.2(3.3)	4.9(11.0)	0.0	1.6(1.9)	3.5(6.1)	3.6(4.4)	22.2(49.7)	0.0	4.9(11.0)	<0.0001
Grazers/scrapers	3.5(5.7)	2.9(3.2)	3.5(6.2)	0.0	0.0	5.5(3.2)	5.2(4.9)	1.3(2.0)	0.0	0.0	0.0	0.0001
Collector-filterers	19.7(14.7)	8.9(11.2)	4.0(3.3)	38.8(30.4)	35.9(29.6)	0.2(0.4)	8.3(15.2)	11.3(10.2)	26.4(30.6)	10.2(13.6)	37.7(35.2)	0.20
Gatherer-collectors	4.2(4.8)	29.6(29.2)	15.0(9.0)	60.4(31.0)	57.5(27.9)	17.1(12.1)	6.9(15.1)	40.9(33.0)	60.4(31.4)	74.5(27.9)	56.2(31.5)	0.004
Shredders	3.7(3.1)	14.3(16.4)	4.5(4.7)	0.8(1.7)	4.5(9.1)	22.5(17.3)	20.0(20.6)	8.4(8.8)	11.9(21.8)	14.8(29.3)	6.1(8.9)	0.03
Predators	9.1(10.0)	1.8(2.5)	3.0(3.9)	0.0	0.0	6.2(3.5)	5.4(5.4)	5.1(3.9)	0.0	0.0	0.0	0.36
other	59.7(17.1)	42.5(20.3)	69.9 (10.9)	0.0	2.0(4.0)	33.9(23.5)	54.2(38.2)	32.9(30.4)	1.3(2.5)	0.5(1.1)	0.0	0.0001

**Table 3:** IndVal procedure results: taxonomic indicators (assignment to FFG) of zones of two segments of Lake Wicko (1 = eulittoral; 2 = infralittoral; 3 = sublittoral). Proportion of randomized trials with indicator value equal to or exceeding the observed indicator value. *p* = (1 + number of runs ≥ observed)/(1 + number of randomized runs). SD–±Standard Deviation.

	Zone with Max. IndVal	Observed	Randomized		*p*
			Mean	SD	
Eastern Segment					
*Limnephilus politus* (Grazers/scraper)	1	55.6	21.4	6.13	0.001
*Ecnomus tenellus* (Predator)	1	32.6	18.4	6.14	0.03
*Planorbarius planorbarius* (Shredder)	1	19.8	8.1	4.20	0.03
*Planorbarius corneus* (Shredder)	1	18.4	9.1	4.44	0.04
*Phryganea grandis* (Grazers/scraper)	2	29.2	10.2	4.77	0.004
*Asellus aquaticus* (Shredder)	2	29.2	18.1	5.43	0.04
*Cladotanytarsus mancus* (other)	3	50.0	19.6	6.91	0.001
Oligochaeta (Gatherer-collector)	3	74.0	45.3	9.96	0.007
Western Segment					
*Ptychoptera* spp. (Collector-filterers)	1	36.3	13.6	5.84	0.005
Chironomidae n. det. (other)	2	42.4	25.5	5.80	0.01

Randomization test for sum of IndVal_max_: observed sum of IndVal_max_ across all variables = 640.53; number of randomization runs = 4999 (none of them with sum of IndVal_max_ ≥ observed value); *p* = 0.0002.

**Table 4:** Annual mean values of bioassessment matrices of the invertebrate community in different depth zones of two segments of Lake Wicko in 2014–2015; *p* = significance of differences between zones (one-way ANOVA). Codes: RICH_t—number of all taxa, CHIR_t—number of taxa of chironomid larvae, EPT_t—number of Ephemeroptera + Plecoptera + Trichoptera larvae, %MolCru—percentage contributions of molluscs and large crustaceans to the total abundance of benthic macroinvertebrates, %OOC—percentage contributions of Oligochaeta/Chironomidae abundance ratio to the total abundance of benthic macroinvertebrates, BMWP—Biological Monitoring Working Party score, ASPT—average score per taxon.

	Western Segment *n* = 30			Eastern Segment *n* = 36			*p*
	Eulittoral	Infralittoral	Sublittoral	Eulittoral	Infralittoral	Sublittoral	
Measures of species richness							
RICH_t	15	22	9	20	25	13	0.0001
CHIR_t	2	5	4	4	5	6	0.40
EPT_t	2	3	0	2	3	0	0.0001
Percentage contributions of indicator groups							
%MolCru	7.3	6.3	0.0	21.7	13.5	0.0	0.0001
%OOC	+	0.2	0.1	0.1	+	0.2	0.005
Biomonitoring indices							
BMWP	54	58	27	60	60	29	0.001
ASPT	3.9	2.8	3.0	3.2	2.9	2.2	0.0001

+ <0.1%.

## Supplementary Materials

The following are available online at https://www.mdpi.com/article/10.3390/ani11092488/s1, Table S1: Physicochemical properties and trophic state indices of water in different depth zones of two segments of Lake Wicko; Table S2: Biological data of the invertebrate community in different depth zones of two segments of Lake Wicko; Table S3: Correlation coefficients (r, after Bonferroni correction) between benthic invertebrates taxa and depths in segments of Lake Wicko; Table S4: Bioassessment indices based on the structure of benthic macroinvertebrates for two segments of Lake Wicko.
