# Supplementary material for: Response of Benthic Fauna to Habitat Heterogeneity in a Shallow Temperate Lake
Source: Animals (Basel). 2021 Aug 25;11(9):2488. doi: 10.3390/ani11092488 (PMC8468703; doi:10.3390/ani11092488)
Supplement: Supplementary file 1 [file animals-11-02488-s001.zip › animals-1302021-SI.pdf]

Table S1. Physicochemical properties and trophic state indices of water in different depth zones of two segments of Lake Wicko.

Depth	Zone	Segment	Month	Year	SD	Temp	pH	DO%	EC	Sal	NO3-	NH4+	PPO43-	TP	Chl-a	Cl-	Na+	K+	Ca2+	Mg^{2+}	TSISD	TSITP	TSIChl
0.3	eulitoral	western	spring	2014	0.20	14.6	8.21	53.6	222	0.099	0.754	0.366	0.035	0.799	89.7	55.3	15.4	1.3	14.4	2.5	83.2	100.5	74.7
0.3	eulitoral	western	summer	2014	0.20	22.3	8.11	60.9	358	0.148	0.947	0.460	0.020	0.452	124.6	52.2	29.3	2.0	17.0	3.0	83.2	92.3	77.9
0.3	eulitoral	western	autumn	2014	0.20	15.1	8.09	46.9	198	0.120	0.221	0.154	0.029	0.648	122.3	42.3	32.4	2.4	20.7	3.4	83.2	97.5	77.8
0.3	eulitoral	western	spring	2015	0.20	13.2	7.81	60.3	235	0.187	0.871	0.372	0.035	0.783	120.4	62.3	12.0	1.7	15.7	3.1	83.2	100.2	77.6
0.3	eulitoral	western	summer	2015	0.20	16.5	8.02	34.9	301	0.155	0.841	0.359	0.031	0.702	226.8	51.6	20.3	2.7	18.3	3.2	83.2	98.7	83.8
0.3	eulitoral	western	autumn	2015	0.20	13.3	8.09	48.9	298	0.148	0.410	0.175	0.029	0.648	226.3	50.6	27.0	4.3	22.0	2.5	83.2	97.5	83.8
0.5	infralitoral	western	spring	2014	0.45	15.6	8.07	60.9	248	0.087	0.547	0.369	0.028	0.628	30.2	58.9	13.7	2.1	14.4	3.7	71.5	97.0	64.0
0.5	infralitoral	western	summer	2014	0.40	19.9	7.95	55.0	354	0.098	0.621	0.419	0.020	0.461	36.5	66.4	23.0	2.4	16.3	3.9	73.2	92.6	65.9
0.5	infralitoral	western	autumn	2014	0.40	8.9	8.03	36.9	333	0.122	0.231	0.156	0.019	0.422	34.7	82.6	29.7	3.1	20.3	4.6	73.2	91.3	65.4
0.5	infralitoral	western	spring	2015	0.35	11.9	8.36	75.3	215	0.098	0.999	0.862	0.030	0.673	37.0	66.4	15.7	1.4	13.0	2.2	75.1	98.0	66.0
0.5	infralitoral	western	summer	2015	0.40	19.8	8.21	77.6	259	0.105	0.874	0.754	0.027	0.619	40.4	71.1	29.5	2.0	18.3	2.7	73.2	96.8	66.9
0.5	infralitoral	western	autumn	2015	0.35	11.9	8.19	39.7	256	0.111	0.321	0.277	0.020	0.452	31.3	75.2	33.4	2.0	24.3	1.5	75.1	92.3	64.4
0.7	infralitoral	western	spring	2014	0.30	15.1	7.91	70.4	256	0.080	0.889	0.214	0.047	1.054	6.2	54.2	32.8	3.2	18.4	3.0	77.3	104.5	48.6
0.7	infralitoral	western	summer	2014	0.25	27.7	8.33	135.9	317	0.100	0.582	0.947	0.017	0.325	7.5	88.5	67.7	8.9	19.2	3.4	80.0	87.5	50.3
0.7	infralitoral	western	autumn	2014	0.30	14.0	8.43	107.1	322	0.100	0.258	0.214	0.025	0.108	7.4	114.2	70.8	5.6	34.5	9.3	77.3	71.6	50.2
0.7	infralitoral	western	spring	2015	0.25	15.5	8.73	113.6	209	0.070	1.296	0.229	0.020	0.846	7.1	53.3	28.8	6.4	25.5	6.3	80.0	101.3	49.9
0.7	infralitoral	western	summer	2015	0.25	15.5	8.68	92.0	456	0.222	0.999	0.032	0.016	0.762	5.4	61.3	40.4	4.4	17.2	3.1	80.0	99.8	47.1
0.7	infralitoral	western	autumn	2015	0.25	14.2	9.24	79.3	512	0.252	1.154	0.547	0.011	0.753	5.4	50.4	32.2	4.3	20.1	2.0	80.0	99.7	47.1
1	sublitoral	western	spring	2014	0.43	14.3	8.59	106.3	209	0.068	0.588	0.323	0.016	0.079	103.3	52.8	30.6	3.0	28.4	0.0	72.3	67.1	76.1
1	sublitoral	western	summer	2014	0.30	26.4	8.88	140.0	207	0.065	0.656	0.263	0.016	0.150	182.7	60.4	36.6	3.6	13.4	1.6	77.3	76.4	81.7
1	sublitoral	western	autumn	2014	0.33	13.7	8.46	105.8	223	0.070	0.235	0.275	0.017	0.046	193.2	53.6	34.8	3.9	32.1	5.5	76.2	59.3	82.2
1	sublitoral	western	spring	2015	0.28	14.8	8.81	115.0	209	0.065	0.814	0.187	0.024	0.680	171.5	50.0	28.0	4.9	20.7	5.4	78.6	98.2	81.1
1	sublitoral	western	summer	2015	0.28	15.6	8.54	82.0	436	0.214	0.712	0.142	0.019	0.963	304.6	56.5	37.6	4.2	27.0	3.6	78.6	103.2	86.7
1	sublitoral	western	autumn	2015	0.25	14.5	8.85	88.7	512	0.248	0.919	0.512	0.020	0.124	85.9	53.4	33.9	3.2	24.0	2.1	80.0	73.6	74.3
3	sublitoral	western	spring	2014	0.40	14.2	8.75	116.2	205	0.070	1.579	0.345	0.020	0.066	95.4	54.2	33.8	3.3	18.7	0.0	73.2	64.6	75.3
3	sublitoral	western	summer	2014	0.20	26.5	8.90	133.5	211	0.067	0.639	0.214	0.017	0.086	15.3	60.1	36.3	3.7	12.5	1.6	83.2	68.4	57.4
3	sublitoral	western	autumn	2014	0.30	14.0	8.68	110.7	217	0.070	0.218	0.365	0.010	0.059	22.7	56.6	36.3	3.9	29.4	5.3	77.3	62.8	61.2
3	sublitoral	western	spring	2015	0.35	13.9	8.80	115.0	210	0.070	6.220	0.175	0.023	0.744	39.9	50.2	27.8	6.3	20.1	5.5	75.1	99.5	66.8

3	sublitoral	western	summer	2015	0.30	15.7	8.66	83.1	429	0.210	1.687	0.114	0.017	0.889	21.5	56.9	37.3	4.1	17.7	2.9	77.3	102.1	60.7
3	sublitoral	western	autumn	2015	0.25	14.2	8.77	87.1	514	0.261	0.749	0.541	0.022	0.044	22.1	54.2	35.1	4.2	18.1	0.2	80.0	58.6	60.9
0.3	eulitoral	eastern	spring	2014	0.20	16.5	8.54	56.3	157	0.040	0.115	0.115	0.003	0.131	23.4	32.6	13.7	1.7	13.7	2.3	83.2	74.5	61.5
0.3	eulitoral	eastern	summer	2014	0.20	28.3	8.60	48.6	201	0.058	0.166	0.087	0.029	0.721	21.4	38.3	15.4	2.0	15.7	2.7	83.2	99.0	60.6
0.3	eulitoral	eastern	autumn	2014	0.20	15.3	7.98	60.3	209	0.120	0.344	0.102	0.017	0.621	20.0	37.8	19.7	2.3	18.7	3.0	83.2	96.9	60.0
0.3	eulitoral	eastern	spring	2015	0.20	15.3	8.11	78.3	154	0.084	0.241	0.236	0.031	0.403	36.2	20.1	13.3	1.5	12.4	1.3	83.2	90.7	65.8
0.3	eulitoral	eastern	summer	2015	0.20	21.3	8.03	60.3	189	0.088	0.253	0.287	0.024	0.532	32.9	21.1	17.0	2.1	15.7	1.7	83.2	94.7	64.9
0.3	eulitoral	eastern	autumn	2015	0.20	14.3	8.24	66.3	199	0.099	0.284	0.154	0.029	0.611	29.3	23.7	21.0	2.1	19.0	2.3	83.2	96.7	63.7
0.5	infralitoral	eastern	spring	2014	0.45	14.9	8.36	80.2	166	0.125	0.359	0.169	0.020	0.222	40.2	30.0	11.1	1.5	11.9	0.9	71.5	82.1	66.8
0.5	infralitoral	eastern	summer	2014	0.40	25.3	7.98	54.9	189	0.154	0.442	0.222	0.023	0.479	39.0	36.9	15.6	2.3	17.0	1.3	73.2	93.1	66.5
0.5	infralitoral	eastern	autumn	2014	0.40	16.3	8.02	74.3	201	0.098	0.281	0.198	0.032	0.679	20.7	42.3	19.3	3.2	19.3	1.9	73.2	98.2	60.3
0.5	infralitoral	eastern	spring	2015	0.35	14.0	8.21	84.6	207	0.119	0.341	0.108	0.030	0.643	48.4	36.4	12.7	1.3	12.3	1.3	75.1	97.4	68.6
0.5	infralitoral	eastern	summer	2015	0.40	26.5	8.20	76.3	229	0.128	0.367	0.122	0.015	0.309	38.3	33.6	16.0	1.6	16.3	1.7	73.2	86.8	66.4
0.5	infralitoral	eastern	autumn	2015	0.30	14.5	8.09	83.3	309	0.058	0.166	0.098	0.020	0.432	43.0	42.1	18.3	2.1	19.0	2.7	77.3	91.7	67.5
0.7	infralitoral	eastern	spring	2014	0.50	13.5	8.64	109.6	188	0.060	0.775	0.147	0.001	0.150	5.5	43.6	27.3	3.1	19.3	1.3	70.0	76.4	47.3
0.7	infralitoral	eastern	summer	2014	0.35	25.7	8.74	129.8	202	0.067	0.562	0.101	0.014	0.051	5.4	55.3	32.8	3.3	15.2	1.3	75.1	61.0	47.1
0.7	infralitoral	eastern	autumn	2014	0.30	13.8	8.41	100.4	214	0.070	0.479	0.569	0.011	0.049	5.2	52.3	34.1	3.9	30.6	5.2	77.3	60.4	46.8
0.7	infralitoral	eastern	spring	2015	0.30	12.9	8.72	114.5	217	0.070	0.460	0.218	0.025	0.793	5.1	49.9	27.9	6.3	20.5	5.4	77.3	100.4	46.5
0.7	infralitoral	eastern	summer	2015	0.20	15.6	8.73	85.3	404	0.200	0.874	0.209	0.014	1.051	6.1	48.5	31.8	3.8	14.8	2.0	83.2	104.5	48.3
0.7	infralitoral	eastern	autumn	2015	0.25	14.5	8.34	81.2	511	0.250	1.610	1.002	0.020	0.436	6.2	52.1	33.5	4.0	41.9	3.3	80.0	91.8	48.5
1	sublitoral	eastern	spring	2014	0.45	15.4	8.89	130.5	189	0.060	0.832	0.321	0.013	0.259	41.0	49.0	29.8	3.2	17.6	0.0	71.5	84.3	67.0
1	sublitoral	eastern	summer	2014	0.32	28.2	8.94	137.2	199	0.060	0.607	0.210	0.012	0.190	46.2	54.6	32.8	3.4	11.4	1.1	76.4	79.8	68.2
1	sublitoral	eastern	autumn	2014	0.30	13.8	8.01	80.3	218	0.070	0.200	0.145	0.037	0.470	74.3	52.6	33.5	3.6	31.3	5.1	77.3	92.9	72.9
1	sublitoral	eastern	spring	2015	0.35	16.2	8.73	126.4	206	0.070	0.387	0.173	0.025	0.822	47.1	50.7	28.1	6.3	20.6	5.4	75.1	100.9	68.4
1	sublitoral	eastern	summer	2015	0.25	16.0	8.59	95.4	398	0.190	1.000	0.074	0.019	0.906	14.2	48.1	31.6	3.6	17.6	2.2	80.0	102.3	56.7
1	sublitoral	eastern	autumn	2015	0.25	14.7	8.68	86.7	511	0.250	1.579	0.604	0.018	0.722	35.8	51.5	34.1	4.4	55.0	4.7	80.0	99.1	65.7
3	sublitoral	eastern	spring	2014	0.50	13.7	8.64	107.4	191	0.060	0.768	0.014	0.012	0.146	6.3	48.7	30.4	3.1	17.1	0.0	70.0	76.0	48.7
3	sublitoral	eastern	summer	2014	0.35	25.1	8.65	122.5	200	0.060	0.620	0.320	0.017	0.235	5.7	53.9	32.4	3.2	16.7	1.2	75.1	82.9	47.7
3	sublitoral	eastern	autumn	2014	0.35	14.1	8.44	102.3	216	0.070	0.226	0.514	0.018	0.242	5.8	51.8	33.5	3.6	31.7	5.4	75.1	83.3	47.9
3	sublitoral	eastern	spring	2015	0.35	14.4	8.67	106.9	211	0.070	0.826	0.160	0.024	0.732	6.1	51.6	28.4	6.3	28.9	5.6	75.1	99.3	48.3
3	sublitoral	eastern	summer	2015	0.25	16.3	8.60	76.1	406	0.200	1.444	0.304	0.014	0.792	6.0	49.8	32.6	3.8	18.6	2.6	80.0	100.4	48.2

3	sublitoral	eastern	autumn	2015	0.25	14.7	8.62	86.7	511	0.250	1.460	0.604	0.022	0.746	6.1	56.0	34.5	3.9	51.8	3.7	80.0	99.5	48.4
5	sublitoral	eastern	spring	2014	0.50	13.8	8.78	114.3	192	0.060	1.701	0.251	0.019	0.241	5.7	49.6	31.2	3.4	17.9	1.7	70.0	83.3	47.6
5	sublitoral	eastern	summer	2014	0.35	25.1	8.79	139.5	283	0.090	0.715	0.039	0.014	0.182	5.9	54.3	32.9	3.3	18.9	1.3	75.1	79.2	48.0
5	sublitoral	eastern	autumn	2014	0.30	13.5	8.02	78.9	212	0.070	0.225	0.367	0.020	0.253	6.1	51.3	33.8	3.8	29.8	5.0	77.3	83.9	48.3
5	sublitoral	eastern	spring	2015	0.35	14.2	8.56	111.3	207	0.070	1.379	0.208	0.021	0.788	6.3	53.2	29.5	6.4	24.0	6.2	75.1	100.3	48.7
5	sublitoral	eastern	summer	2015	0.30	16.1	8.53	80.4	406	0.200	0.874	0.095	0.019	0.911	6.1	49.3	32.3	3.7	17.0	2.4	77.3	102.4	48.4
5	sublitoral	eastern	autumn	2015	0.30	14.0	9.45	79.3	512	0.250	2.643	0.995	0.033	0.452	6.3	52.7	35.5	4.1	49.2	4.4	77.3	92.3	48.6

Table S2. Biological data of the invertebrate community in different depth zones of two segments of Lake Wicko.

**Depth**	**Zone**	**Segment**	**Month**	**Year**	Oligochaeta	*Erpobdella octoculata*	*Glossiphonia complanata*	*Helobdella stagnalis*	*Asellus aquaticus*	*Sialis lutaria*	*Ecnomus tenellus*	*Phryganea grandis*	*Limnephilus politus*	*Ptychoptera* sp.	*Chironomidae n.det.*	*Einfeldia carbonaria*	*Tanytarsus gregarius*	*Chironomus* sp.	*Cryptochironomus conjugens*	*Bezzia* sp.	*Polypedilum nubeculosus*	*Polypedilum scalaeum*	*Cladotanytarsus mancus*	*Valvata piscinalis*	*Planorbarius corneus*	*Planorbis planorbis*	*Theodoxus fluviatilis*	*Sphaerium corneum*	*Unio pictorumm*
0.3	eulitoral	western	spring	2014	0.0	0.0	0.0	0.0	0.0	0.0	2.6	0.0	0.5	2.9	4.7	0.0	0.0	0.0	0.0	0.0	0.0	0.0	0.0	0.0	0.0	0.0	0.0	0.0	1.9
0.3	eulitoral	western	summer	2014	0.0	0.0	0.0	0.0	0.0	0.0	0.0	0.0	2.9	93.4	152.3	2.3	0.0	0.0	0.0	0.0	0.0	0.0	0.0	0.0	0.0	2.2	1.2	0.0	0.0
0.3	eulitoral	western	autumn	2014	11.2	5.6	0.0	0.0	12.6	0.0	0.0	0.0	35.3	0.0	156.3	0.0	0.0	0.0	0.0	1.2	0.0	0.0	0.0	0.0	0.0	0.0	0.0	0.0	0.0
0.3	eulitoral	western	spring	2015	25.6	0.0	0.0	0.0	15.6	0.0	0.0	0.0	0.0	28.6	112.9	0.0	0.0	0.0	0.0	0.0	0.0	0.0	0.0	0.0	0.0	0.0	0.0	0.0	15.6
0.3	eulitoral	western	summer	2015	0.0	0.0	0.0	0.0	0.0	0.0	24.6	0.0	0.8	0.0	322.0	0.0	0.0	0.0	0.0	0.0	0.0	0.0	0.0	0.0	0.0	0.0	0.0	9.6	2.3
0.3	eulitoral	western	autumn	2015	14.6	0.0	0.0	0.0	0.0	0.0	50.3	0.0	0.0	35.6	89.3	0.0	0.0	0.0	0.0	2.2	0.0	0.0	0.0	0.0	10.3	0.0	0.0	0.0	0.0
0.5	infralitoral	western	spring	2014	0.0	1.2	0.0	0.0	0.0	0.0	0.0	0.0	0.0	0.0	54.9	0.0	0.0	0.0	0.0	52.1	0.0	0.0	0.0	0.0	0.0	0.0	0.0	0.0	0.0
0.5	infralitoral	western	summer	2014	25.3	0.0	0.0	0.0	0.0	0.0	2.6	0.0	0.0	0.0	84.3	0.0	0.0	36.1	0.0	0.0	0.0	0.0	0.0	1.3	3.1	0.0	0.0	0.0	0.0
0.5	infralitoral	western	autumn	2014	0.0	0.0	11.3	0.0	0.0	0.0	2.9	0.0	5.9	0.0	0.0	0.0	0.0	174.3	0.0	0.0	0.0	0.0	0.0	0.0	0.0	0.0	0.0	0.0	2.3
0.5	infralitoral	western	spring	2015	11.3	0.0	0.0	0.0	23.6	0.0	0.0	0.0	11.3	35.6	54.4	0.0	0.0	0.0	0.0	0.0	2.3	0.0	0.0	3.6	0.0	0.0	0.0	0.0	2.3
0.5	infralitoral	western	summer	2015	0.0	0.0	0.0	0.0	0.0	1.3	0.0	0.0	0.0	36.0	87.9	0.0	0.0	0.0	26.3	22.3	0.0	0.0	3.3	0.0	0.0	0.0	0.0	0.0	5.9
0.5	infralitoral	western	autumn	2015	44.6	0.0	0.0	0.0	0.0	0.0	0.0	11.3	0.0	0.0	108.9	0.0	0.0	0.0	0.0	0.0	0.0	0.0	0.0	6.9	0.0	0.0	0.0	0.0	5.6
0.7	infralitoral	western	spring	2014	2.3	0.0	0.0	0.0	0.0	0.0	2.3	0.0	0.0	0.0	14.1	0.0	0.0	0.0	0.0	0.0	0.0	0.0	0.0	0.0	0.0	2.3	0.0	0.0	0.0
0.7	infralitoral	western	summer	2014	15.8	0.0	0.0	0.0	0.0	0.0	0.0	0.0	2.3	2.3	32.3	0.0	0.0	0.0	0.0	0.0	0.0	0.0	0.0	0.0	0.0	0.0	3.6	0.0	0.0
0.7	infralitoral	western	autumn	2014	5.9	1.3	0.0	1.0	0.0	0.0	0.0	0.0	0.0	0.0	56.3	0.0	0.0	0.0	15.9	0.0	0.0	0.0	0.0	0.0	0.0	0.0	0.0	0.0	8.5
0.7	infralitoral	western	spring	2015	12.3	0.0	0.0	0.0	12.3	5.6	0.0	0.0	0.0	1.3	89.3	0.0	0.0	0.0	0.0	0.0	0.0	0.0	0.0	0.0	0.0	0.0	0.0	0.0	4.5
0.7	infralitoral	western	summer	2015	1.4	0.0	0.0	0.0	0.0	0.0	0.0	0.3	0.0	5.9	91.7	0.0	0.0	0.0	0.0	0.0	0.0	0.0	0.0	0.0	0.0	0.0	0.0	0.0	0.0
0.7	infralitoral	western	autumn	2015	12.3	0.0	0.0	0.0	0.0	0.0	0.0	12.0	5.3	0.0	69.2	0.0	0.0	3.5	0.0	0.0	0.0	0.0	0.0	0.0	0.0	0.0	0.0	0.0	0.0
1	sublitoral	western	spring	2014	214.9	0.0	0.0	0.0	0.0	0.0	0.0	0.0	0.0	29.7	0.0	0.0	29.7	22.2	0.0	0.0	0.0	0.0	0.0	0.0	0.0	0.0	0.0	0.0	0.0

1	sublitoral	western	summer	2014	44.4	0.0	0.0	0.0	0.0	0.0	0.0	0.0	0.0	96.3	0.0	0.0	14.8	0.0	0.0	0.0	0.0	0.0	0.0	7.4	0.0	0.0	0.0	0.0	0.0
1	sublitoral	western	autumn	2014	14.8	0.0	0.0	0.0	0.0	0.0	0.0	0.0	0.0	103.7	0.0	0.0	7.4	214.8	0.0	0.0	0.0	0.0	0.0	0.0	0.0	0.0	0.0	0.0	29.7
1	sublitoral	western	spring	2015	7.4	0.0	0.0	0.0	0.0	0.0	0.0	0.0	0.0	81.5	0.0	0.0	0.0	0.0	0.0	0.0	0.0	0.0	0.0	0.0	0.0	0.0	0.0	0.0	0.0
1	sublitoral	western	summer	2015	111.1	0.0	0.0	0.0	0.0	0.0	0.0	0.0	0.0	133.4	0.0	0.0	0.0	118.5	7.4	0.0	0.0	0.0	0.0	0.0	0.0	0.0	0.0	0.0	0.0
1	sublitoral	western	autumn	2015	22.2	0.0	0.0	0.0	0.0	0.0	0.0	0.0	0.0	0.0	0.0	0.0	14.8	355.6	0.0	0.0	0.0	0.0	0.0	0.0	0.0	0.0	0.0	0.0	0.0
3	sublitoral	western	spring	2014	74.1	0.0	0.0	0.0	0.0	0.0	0.0	0.0	0.0	88.9	0.0	0.0	0.0	0.0	0.0	0.0	0.0	0.0	0.0	0.0	0.0	0.0	0.0	0.0	0.0
3	sublitoral	western	summer	2014	0.0	0.0	0.0	0.0	0.0	0.0	0.0	0.0	0.0	74.1	0.0	0.0	14.8	0.0	0.0	0.0	0.0	0.0	0.0	0.0	0.0	0.0	0.0	0.0	0.0
3	sublitoral	western	autumn	2014	14.8	0.0	0.0	0.0	0.0	0.0	0.0	0.0	0.0	0.0	0.0	0.0	0.0	8474.1	281.5	0.0	0.0	0.0	0.0	0.0	0.0	0.0	0.0	0.0	0.0
3	sublitoral	western	spring	2015	0.0	0.0	0.0	0.0	0.0	0.0	0.0	0.0	0.0	0.0	0.0	0.0	0.0	0.0	0.0	0.0	0.0	0.0	0.0	0.0	0.0	0.0	0.0	0.0	0.0
3	sublitoral	western	summer	2015	29.6	0.0	0.0	0.0	0.0	0.0	0.0	0.0	0.0	74.1	44.4	0.0	0.0	296.3	0.0	0.0	0.0	0.0	0.0	0.0	0.0	0.0	0.0	0.0	0.0
3	sublitoral	western	autumn	2015	0.0	0.0	0.0	0.0	0.0	0.0	0.0	0.0	0.0	163.0	0.0	0.0	0.0	340.7	0.0	0.0	0.0	0.0	0.0	0.0	0.0	0.0	148.1	0.0	0.0
0.3	eulitoral	eastern	spring	2014	20.3	0.0	3.6	0.0	13.3	0.2	2.3	0.0	1.3	2.3	43.6	5.3	0.0	0.0	12.3	0.0	0.0	0.0	0.0	0.0	0.0	0.0	1.2	0.0	0.0
0.3	eulitoral	eastern	summer	2014	0.0	1.3	1.5	0.0	15.3	0.6	1.7	0.0	5.6	1.3	91.5	0.0	0.0	0.0	0.0	0.0	0.0	0.0	0.0	0.0	1.3	2.2	0.0	0.0	0.0
0.3	eulitoral	eastern	autumn	2014	12.3	0.0	3.6	0.0	20.3	0.0	5.6	0.0	12.3	12.5	14.9	14.8	0.0	0.0	0.0	0.0	2.3	0.0	0.0	10.0	0.0	0.0	0.0	0.0	0.0
0.3	eulitoral	eastern	spring	2015	14.3	0.0	6.9	0.0	21.0	11.0	2.3	0.0	10.3	22.2	62.4	0.0	0.0	0.0	12.3	19.3	0.0	0.0	0.0	0.0	0.0	0.0	0.0	0.0	5.3
0.3	eulitoral	eastern	summer	2015	20	3.5	0	14.6	0	0	0	0	6.4	67.2	88.9	0.0	0.0	0.0	0.5	0.0	0.0	0.0	0.0	12.7	0.0	5.7	0.0	0.0	1.5
0.3	eulitoral	eastern	autumn	2015	12.6	0.0	0.0	0.0	45.3	0.0	0.0	0.0	11.9	12.9	0.0	0.0	0.0	0.0	45.2	0.0	0.0	0.0	0.0	15.6	6.8	0.0	12.4	0.0	2.6
0.5	infralitoral	eastern	spring	2014	0.0	2.3	0.0	0.0	22.9	12.7	0.0	8.6	3.4	0.0	0.0	19.7	0.0	0.0	0.0	0.0	0.0	0.0	2.1	11.3	2.3	0.0	0.0	0.3	1.6
0.5	infralitoral	eastern	summer	2014	3.9	0.8	1.9	3.1	27.0	1.3	0.0	2.1	3.3	99.3	0.0	0.0	0.0	91.5	0.0	0.0	0.0	0.0	0.0	1.1	0.0	0.0	0.0	0.0	0.0
0.5	infralitoral	eastern	autumn	2014	0.0	0.0	0.0	0.0	22.3	0.0	22.3	22.0	21.7	0.0	348.2	0.0	0.0	0.0	0.0	0.0	0.0	0.0	0.0	5.6	7.9	0.0	0.0	0.0	2.3
0.5	infralitoral	eastern	spring	2015	0.0	0.0	0.0	0.0	62.3	0.0	4.3	0.0	11.8	0.0	222.7	0.0	0.0	0.0	0.0	0.0	0.0	0.0	0.0	0.0	0.0	0.0	8.9	12.3	4.6
0.5	infralitoral	eastern	summer	2015	0	0	0	0	12.9	4.3	3	0	2.6	0.0	198.3	0.0	0.0	0.0	0.0	0.0	0.0	0.0	0.0	0.0	0.0	3.0	2.2	0.0	0.0
0.5	infralitoral	eastern	autumn	2015	3.3	2.3	0.0	0.0	26.3	0.0	12.3	0.0	2.9	0.0	478.3	0.0	0.5	0.0	0.0	0.0	0.0	0.0	10.0	0.0	0.0	0.0	5.4	0.0	0.0
0.7	infralitoral	eastern	spring	2014	2.2	0.5	2.3	2.3	25.6	0.0	6.5	0.0	5.8	0.4	55.1	0.0	0.0	0.0	0.0	0.0	0.0	0.0	0.0	0.0	0.0	0.0	0.0	4.6	0.0
0.7	infralitoral	eastern	summer	2014	4.3	0.0	0.5	0.0	0.3	0.0	24.6	1.4	1.6	2.6	320.2	0.0	0.0	14.4	0.0	0.0	0.0	0.0	0.0	0.0	0.0	0.0	0.0	0.0	0.0
0.7	infralitoral	eastern	autumn	2014	2.7	1.5	2.9	0.0	35.3	0.0	5.6	0.0	0.0	112.8	0.0	0.0	0.0	212.9	0.0	0.0	0.0	0.0	0.0	25.6	0.0	0.0	0.0	0.0	0.0

0.7	infralitoral	eastern	spring	2015	12.6	2.3	1.3	0.0	27.7	0.0	54.9	0.0	9.2	136.5	248.9	0.0	0.0	189.3	0.0	0.0	0.0	0.0	0.0	0.0	0.0	0.0	0.0	4.6	0.0
0.7	infralitoral	eastern	summer	2015	9.9	0.0	0.6	0.0	36.1	2.5	0.0	0.0	0.2	65.3	120.0	0.0	0.0	267.3	31.0	0.0	0.0	0.0	0.0	0.0	0.0	0.0	0.0	0.0	0.0
0.7	infralitoral	eastern	autumn	2015	89.3	12.3	0.0	0.0	0.0	0.0	0.0	0.0	0.0	0.0	0.0	0.0	0.0	727.3	0.0	0.0	0.0	0.0	0.0	0.0	0.0	0.0	0.0	12.4	0.0
1	sublitoral	eastern	spring	2014	1525.9	0.0	0.0	0.0	0.0	0.0	0.0	0.0	0.0	237.0	0.0	0.0	0.0	0.0	0.0	14.8	0.0	0.0	0.0	0.0	0.0	0.0	0.0	0.0	0.0
1	sublitoral	eastern	summer	2014	948.1	0.0	0.0	0.0	0.0	0.0	0.0	0.0	0.0	651.9	0.0	0.0	118.5	0.0	0.0	29.6	0.0	0.0	0.0	0.0	0.0	0.0	0.0	0.0	0.0
1	sublitoral	eastern	autumn	2014	0.0	0.0	0.0	0.0	0.0	0.0	0.0	0.0	0.0	0.0	0.0	0.0	0.0	59.3	0.0	74.1	0.0	0.0	0.0	0.0	0.0	0.0	0.0	0.0	0.0
1	sublitoral	eastern	spring	2015	0.0	0.0	0.0	0.0	0.0	0.0	0.0	0.0	0.0	0.0	0.0	0.0	0.0	0.0	0.0	0.0	0.0	0.0	0.0	0.0	0.0	0.0	0.0	0.0	0.0
1	sublitoral	eastern	summer	2015	88.9	0.0	0.0	0.0	0.0	0.0	0.0	0.0	0.0	637.0	59.3	0.0	14.8	0.0	0.0	0.0	0.0	0.0	0.0	14.8	0.0	0.0	0.0	133.3	0.0
1	sublitoral	eastern	autumn	2015	59.3	0.0	0.0	0.0	0.0	0.0	0.0	0.0	0.0	0.0	0.0	0.0	0.0	385.2	0.0	0.0	0.0	0.0	0.0	0.0	0.0	0.0	0.0	0.0	0.0
3	sublitoral	eastern	spring	2014	0.0	0.0	0.0	0.0	0.0	0.0	0.0	0.0	0.0	0.0	0.0	14.8	14.8	0.0	0.0	0.0	0.0	44.4	0.0	0.0	0.0	0.0	0.0	0.0	0.0
3	sublitoral	eastern	summer	2014	29.6	0.0	0.0	0.0	0.0	0.0	0.0	0.0	0.0	44.4	0.0	0.0	44.4	0.0	0.0	0.0	0.0	0.0	0.0	0.0	0.0	0.0	0.0	0.0	0.0
3	sublitoral	eastern	autumn	2014	88.9	0.0	0.0	0.0	0.0	0.0	0.0	0.0	0.0	14.8	0.0	0.0	651.9	533.3	0.0	0.0	0.0	0.0	0.0	0.0	0.0	0.0	0.0	0.0	0.0
3	sublitoral	eastern	spring	2015	44.4	0.0	0.0	0.0	0.0	0.0	0.0	0.0	0.0	59.3	0.0	0.0	29.6	0.0	0.0	0.0	177.8	29.6	0.0	0.0	0.0	0.0	0.0	0.0	0.0
3	sublitoral	eastern	summer	2015	148.1	0.0	0.0	0.0	0.0	0.0	0.0	0.0	0.0	29.6	0.0	0.0	0.0	0.0	0.0	0.0	355.6	0.0	14.8	0.0	0.0	0.0	0.0	0.0	0.0
3	sublitoral	eastern	autumn	2015	59.3	0.0	0.0	0.0	0.0	0.0	0.0	0.0	0.0	0.0	0.0	0.0	0.0	0.0	0.0	0.0	266.7	0.0	0.0	0.0	0.0	0.0	0.0	0.0	0.0
5	sublitoral	eastern	spring	2014	163.0	0.0	0.0	0.0	0.0	0.0	0.0	0.0	0.0	88.9	0.0	0.0	14.8	0.0	0.0	14.8	0.0	29.6	0.0	0.0	0.0	0.0	0.0	0.0	0.0
5	sublitoral	eastern	summer	2014	14.8	0.0	0.0	0.0	0.0	0.0	0.0	0.0	0.0	118.5	0.0	0.0	0.0	0.0	0.0	0.0	0.0	0.0	0.0	0.0	0.0	0.0	0.0	0.0	0.0
5	sublitoral	eastern	autumn	2014	29.6	0.0	0.0	0.0	0.0	0.0	0.0	0.0	0.0	0.0	0.0	0.0	0.0	44.4	0.0	0.0	0.0	0.0	0.0	0.0	0.0	0.0	0.0	0.0	29.6
5	sublitoral	eastern	spring	2015	74.1	0.0	0.0	0.0	0.0	0.0	0.0	0.0	0.0	0.0	0.0	0.0	14.8	0.0	0.0	0.0	14.8	29.6	0.0	0.0	0.0	0.0	0.0	0.0	0.0
5	sublitoral	eastern	summer	2015	29.6	0.0	0.0	0.0	0.0	0.0	0.0	0.0	0.0	118.5	0.0	0.0	0.0	0.0	0.0	0.0	0.0	0.0	0.0	0.0	0.0	0.0	0.0	0.0	0.0
5	sublitoral	eastern	autumn	2015	14.8	0.0	0.0	0.0	0.0	0.0	0.0	0.0	0.0	0.0	0.0	0.0	74.1	44.4	0.0	0.0	0.0	0.0	0.0	0.0	0.0	0.0	0.0	0.0	0.0

Table S3. Correlation coefficients (r, after Bonferroni correction) between benthic invertebrates taxa and depth in segments of Lake Wicko.

Taxa	all sites	western segment	eastern segment
Polypedilum scalaeum	**0.416**	0.000	**0.434**
Polypedilum nubculosum	0.232	-0.114	0.222
Tanatyrus mancus	0.185	0.108	0.175
Chironomus sp.	0.134	**0.385**	-0.098
Theodoxus fluviatilis	0.111	**0.358**	-0.253
Cryptochironomus conjugens	0.086	**0.345**	-0.239
Unio pictorum	0.046	-0.190	0.237
Tanytarsus gregarius	0.039	-0.114	0.019
Procladius spp.	0.038	**0.410**	-0.057
Ptychoptera spp.	0.034	**0.396**	-0.058
Oligochaeta	0.008	0.059	-0.039
Sphaerium corneus	-0.063	-0.152	-0.103
Einfeldia carbonaria	-0.065	-0.152	-0.124
Bezzia sp.	-0.085	-0.161	-0.083
Helobdella stagnalis	-0.126	-0.076	-0.182
Phryganea grandis	-0.158	-0.137	-0.179
Sialis lutaria	-0.182	-0.101	-0.249
Planorbis planorbis	-0.189	-0.163	-0.227
Erpobdella octoculata	-0.190	-0.188	-0.237
Planorbarius corneus	-0.195	-0.180	-0.232
Glossiphonia complanata	-0.215	-0.114	**-0.365**
Ecnomus tenellus	-0.225	-0.229	-0.262
Valvata piscinalis	-0.226	-0.143	**-0.317**
Limnophilus politus	**-0.320**	-0.232	**-0.468**
Asellus aquaticus	**-0.361**	-0.249	**-0.530**
Chironomidae n.det.	**-0.387**	**-0.473**	**-0.393**

Table S4. Bioassessment indices based on the structure of benthic invertebrates for two segments of Lake Wicko.

**Depth**	**Zones**	**Segment**	**Month**	**Year**	**Total**	**H'**	**J'**	RICHtot	BMWP	ASPT	CHIR_t	EPT	MOLCRU_t	**total EPT**	**oligochaeta/ chironomidae**	**OOC**	**Gr/Sc**	**FF**	**GaCol**	**Shr**	**Pred**	**other**
0.3	eulitoral	western	spring	2014	12.6	1.445	0.848	5	23	5.8	1	2	1.9	3.1	4.7	0.00	0.5	4.8	0.0	0.0	2.6	4.7
0.3	eulitoral	western	summer	2014	254.3	0.835	0.384	6	20	6.7	2	1	3.4	2.9	154.6	0.00	2.9	93.4	0.0	5.7	0.0	152.3
0.3	eulitoral	western	autumn	2014	222.2	0.974	0.441	6	18	3.6	1	1	12.6	35.3	168.7	0.07	35.3	0.0	11.2	13.8	5.6	156.3
0.3	eulitoral	western	spring	2015	198.3	1.264	0.708	5	24	4.8	1	0	31.2	0.0	138.5	0.23	0.0	44.2	25.6	15.6	0.0	112.9
0.3	eulitoral	western	summer	2015	359.3	0.425	0.306	5	20	4.0	1	2	11.9	25.4	322.0	0.00	0.8	11.9	0.0	0.0	24.6	322.0
0.3	eulitoral	western	autumn	2015	202.3	1.403	0.678	6	15	3.8	1	1	10.3	50.3	106.1	0.16	0.0	35.6	14.6	12.5	50.3	89.3
0.5	infralitoral	western	spring	2014	108.2	0.746	0.703	3	6	3.0	1	0	0.0	0.0	107.0	0.00	0.0	0.0	0.0	52.1	1.2	54.9
0.5	infralitoral	western	summer	2014	152.7	1.156	0.529	6	19	4.8	2	3	4.4	2.6	145.7	0.21	0.0	0.0	61.4	4.4	2.6	84.3
0.5	infralitoral	western	autumn	2014	196.7	0.491	0.327	5	26	5.2	1	3	2.3	8.8	174.3	0.00	5.9	2.3	174.3	0.0	14.2	0.0
0.5	infralitoral	western	spring	2015	144.4	1.632	0.639	8	22	3.7	2	0	29.5	11.3	68.0	0.20	11.3	37.9	13.6	27.2	0.0	54.4
0.5	infralitoral	western	summer	2015	183.0	1.426	0.594	7	30	10.0	3	3	5.9	0.0	136.5	0.00	0.0	41.9	26.3	22.3	1.3	91.2
0.5	infralitoral	western	autumn	2015	177.3	1.057	0.576	5	21	5.3	1	0	12.5	11.3	153.5	0.41	11.3	5.6	44.6	6.9	0.0	108.9
0.7	infralitoral	western	spring	2014	21.0	0.994	0.676	4	15	3.8	1	1	2.3	2.3	16.4	0.16	0.0	0.0	2.3	2.3	2.3	14.1
0.7	infralitoral	western	summer	2014	56.3	1.112	0.608	5	19	4.8	1	1	3.6	2.3	48.1	0.49	2.3	2.3	15.8	3.6	0.0	32.3
0.7	infralitoral	western	autumn	2014	88.9	1.114	0.508	6	11	2.2	2	0	8.5	0.0	78.1	0.08	0.0	8.5	21.8	0.0	2.3	56.3
0.7	infralitoral	western	spring	2015	125.3	1.003	0.454	6	23	3.8	1	0	16.8	0.0	101.6	0.14	0.0	5.8	12.3	12.3	5.6	89.3
0.7	infralitoral	western	summer	2015	99.3	0.319	0.344	4	10	2.5	1	1	0.0	0.3	93.1	0.02	0.3	5.9	1.4	0.0	0.0	91.7
0.7	infralitoral	western	autumn	2015	102.3	1.039	0.566	5	12	3.0	2	2	0.0	17.3	85.0	0.17	17.3	0.0	15.8	0.0	0.0	69.2
1	sublitoral	western	spring	2014	296.4	0.888	0.608	2	5	2.5	2	0	0	0.0	296.4	2.64	0.0	29.7	266.7	0.0	0.0	0.0
1	sublitoral	western	summer	2014	162.9	1.023	0.696	3	9	3.0	1	0	0	0.0	155.5	0.40	0.0	96.3	59.2	7.4	0.0	0.0
1	sublitoral	western	autumn	2014	370.4	1.082	0.590	3	12	4.0	2	0	0	0.0	340.7	0.05	0.0	133.4	237.0	0.0	0.0	0.0
1	sublitoral	western	spring	2015	88.9	0.287	0.666	2	5	2.5	0	0	0	0.0	88.9	0.09	0.0	81.5	7.4	0.0	0.0	0.0
1	sublitoral	western	summer	2015	370.4	1.172	0.807	2	5	2.5	2	0	0	0.0	370.4	0.43	0.0	133.4	237.0	0.0	0.0	0.0
1	sublitoral	western	autumn	2015	392.6	0.376	0.485	2	5	2.5	2	0	0	0.0	392.6	0.06	0.0	0.0	392.6	0.0	0.0	0.0
3	sublitoral	western	spring	2014	163.0	0.689	0.996	2	5	2.5	0	0	0	0.0	163.0	0.83	0.0	88.9	74.1	0.0	0.0	0.0
3	sublitoral	western	summer	2014	88.9	0.450	0.784	1	3	3.0	1	0	0	0.0	88.9	0.00	0.0	74.1	14.8	0.0	0.0	0.0
3	sublitoral	western	autumn	2014	8770.4	0.154	0.389	2	5	2.5	2	0	0	0.0	8770.4	0.00	0.0	0.0	8770.4	0.0	0.0	0.0

3	sublitoral	western	spring	2015	0.0	0.000	0.000	0	0	0.0	0	0	0	0.0	0.0	0.00	0.0	0.0	0.0	0.0	0.0	0.0
3	sublitoral	western	summer	2015	444.4	0.980	0.666	2	5	2.5	2	0	0	0.0	444.4	0.07	0.0	74.1	325.9	0.0	0.0	44.4
3	sublitoral	western	autumn	2015	651.8	1.022	0.927	2	9	4.5	1	0	0	0.0	503.7	0.00	0.0	163.0	340.7	148.1	0.0	0.0
0.3	eulitoral	eastern	spring	2014	105.7	1.742	0.519	11	34	3.4	3	2	14.5	3.6	83.8	0.32	1.3	2.3	32.6	19.8	6.1	43.6
0.3	eulitoral	eastern	summer	2014	122.3	0.975	0.265	10	36	3.6	1	2	18.8	7.3	92.8	0.00	5.6	1.3	0.0	18.8	5.1	91.5
0.3	eulitoral	eastern	autumn	2014	108.6	2.167	0.873	11	33	4.0	3	2	30.3	17.9	56.8	0.28	12.3	12.5	14.6	45.1	9.2	14.9
0.3	eulitoral	eastern	spring	2015	187.3	2.076	0.725	10	39	3.6	2	2	26.3	12.6	130.5	0.12	10.3	27.5	26.6	40.3	20.2	62.4
0.3	eulitoral	eastern	summer	2015	221.0	1.600	0.495	11	38	3.6	2	1	19.9	6.4	176.6	0.13	6.4	68.7	20.5	18.4	18.1	88.9
0.3	eulitoral	eastern	autumn	2015	165.3	1.908	0.749	9	40	4.2	1	1	82.7	11.9	70.7	0.22	11.9	15.5	57.8	80.1	0.0	0.0
0.5	infralitoral	eastern	spring	2014	87.2	1.962	0.647	11	39	3.9	2	2	38.4	12.0	21.8	0.00	12.0	1.9	0.0	56.2	15.0	2.1
0.5	infralitoral	eastern	summer	2014	235.3	1.319	0.340	8	33	2.8	1	2	28.1	5.4	194.7	0.02	5.4	99.3	95.4	28.1	7.1	0.0
0.5	infralitoral	eastern	autumn	2014	452.3	0.943	0.321	8	34	2.9	1	3	38.1	66.0	348.2	0.00	43.7	2.3	0.0	35.8	22.3	348.2
0.5	infralitoral	eastern	spring	2015	326.9	1.036	0.403	7	38	4.0	1	2	88.1	16.1	222.7	0.00	11.8	16.9	0.0	71.2	4.3	222.7
0.5	infralitoral	eastern	summer	2015	226.3	0.565	0.251	6	34	2.5	1	2	18.1	5.6	198.3	0.00	2.6	0.0	0.0	18.1	7.3	198.3
0.5	infralitoral	eastern	autumn	2015	541.3	0.551	0.193	9	33	4.0	3	2	31.7	15.2	492.1	0.01	2.9	0.0	3.8	31.7	14.6	488.3
0.7	infralitoral	eastern	spring	2014	105.3	1.446	0.424	10	36	4.0	1	2	30.2	12.3	57.7	0.04	5.8	5.0	2.2	25.6	11.6	55.1
0.7	infralitoral	eastern	summer	2014	369.9	0.578	0.198	9	24	2.6	2	3	0.3	27.6	341.5	0.01	3.0	2.6	18.7	0.3	25.1	320.2
0.7	infralitoral	eastern	autumn	2014	399.3	1.233	0.429	8	26	2.5	1	1	60.9	5.6	328.4	0.01	0.0	112.8	215.6	60.9	10.0	0.0
0.7	infralitoral	eastern	spring	2015	687.3	1.571	0.481	10	30	3.1	2	2	32.3	64.1	587.3	0.02	9.2	141.1	201.9	27.7	58.5	248.9
0.7	infralitoral	eastern	summer	2015	532.9	1.397	0.449	9	21	4.7	3	1	36.1	0.2	493.5	0.02	0.2	65.3	308.2	36.1	3.1	120.0
0.7	infralitoral	eastern	autumn	2015	841.3	0.488	0.407	5	18	3.3	1	0	12.4	0.0	816.6	0.12	0.0	12.4	816.6	0.0	12.3	0.0
1	sublitoral	eastern	spring	2014	1777.7	0.440	0.517	3	9	3.0	0	0	0.0	0.0	1777.7	6.06	0.0	237.0	1525.9	14.8	0.0	0.0
1	sublitoral	eastern	summer	2014	1748.1	0.951	0.647	3	9	3.0	1	0	0.0	0.0	1748.1	1.19	0.0	651.9	1066.6	29.6	0.0	0.0
1	sublitoral	eastern	autumn	2014	133.4	0.687	0.994	2	7	3.5	1	0	0.0	0.0	133.4	0.00	0.0	0.0	59.3	74.1	0.0	0.0
1	sublitoral	eastern	spring	2015	0.0	0.000	0.000	0	0	0.0	0	0	0.0	0.0	0.0	0.00	0.0	0.0	0.0	0.0	0.0	0.0
1	sublitoral	eastern	summer	2015	948.1	1.068	0.485	4	13	3.3	2	0	148.1	0.0	800.0	0.13	0.0	770.3	103.7	14.8	0.0	59.3
1	sublitoral	eastern	autumn	2015	444.5	0.393	0.741	2	5	2.5	1	0	0.0	0.0	444.5	0.15	0.0	0.0	444.5	0.0	0.0	0.0
3	sublitoral	eastern	spring	2014	74.0	0.950	0.862	1	3	3.0	2	0	0.0	0	74.0	0.00	0.0	0.0	14.8	59.2	0.0	0.0
3	sublitoral	eastern	summer	2014	118.4	1.082	0.984	2	5	2.5	1	0	0.0	0	118.4	0.33	0.0	44.4	74.0	0.0	0.0	0.0
3	sublitoral	eastern	autumn	2014	1288.9	0.946	0.644	2	5	2.5	2	0	0.0	0	1288.9	0.07	0.0	14.8	1274.1	0.0	0.0	0.0

3	sublitoral	eastern	spring	2015	340.7	1.334	0.759	2	5	2.5	2	0	0.0	0	340.7	0.15	0.0	59.3	251.8	29.6	0.0	0.0
3	sublitoral	eastern	summer	2015	548.1	0.889	0.608	2	5	2.5	2	0	0.0	0	548.1	0.37	0.0	29.6	503.7	0.0	0.0	14.8
3	sublitoral	eastern	autumn	2015	326.0	0.474	0.803	2	5	2.5	1	0	0.0	0	326.0	0.22	0.0	0.0	326.0	0.0	0.0	0.0
5	sublitoral	eastern	spring	2014	311.1	1.210	0.671	3	9	3.0	1	0	0.0	0	311.1	1.10	0.0	88.9	177.8	44.4	0.0	0.0
5	sublitoral	eastern	summer	2014	133.3	0.349	0.709	2	5	2.5	0	0	0.0	0	133.3	0.12	0.0	118.5	14.8	0.0	0.0	0.0
5	sublitoral	eastern	autumn	2014	103.6	1.079	0.981	3	12	4.0	1	0	29.6	0	74.0	0.67	0.0	29.6	74.0	0.0	0.0	0.0
5	sublitoral	eastern	spring	2015	133.3	1.149	0.789	2	5	2.5	2	0	0.0	0	133.3	1.25	0.0	0.0	103.7	29.6	0.0	0.0
5	sublitoral	eastern	summer	2015	148.1	0.500	0.825	2	5	2.5	0	0	0.0	0	148.1	0.25	0.0	118.5	29.6	0.0	0.0	0.0
5	sublitoral	eastern	autumn	2015	133.3	0.937	0.851	2	5	2.5	2	0	0.0	0	133.3	0.12	0.0	0.0	133.3	0.0	0.0	0.0
